# Identification of restrictive molecules involved in oncolytic virotherapy using genome-wide CRISPR screening

**DOI:** 10.1186/s13045-024-01554-5

**Published:** 2024-05-23

**Authors:** Yiye Zhong, Huangying Le, Xue Zhang, Yao Dai, Fang Guo, Xiaojuan Ran, Guohong Hu, Qi Xie, Dawei Wang, Yujia Cai

**Affiliations:** 1 Key Laboratory of Systems Biomedicine (Ministry of Education), Shanghai Center for Systems Biomedicine, Shanghai Jiao Tong University, Shanghai, 200240 China; 2 School of Life Sciences, Westlake University, Hangzhou, 310024 China; 3 CAS Key Laboratory of Tissue Microenvironment and Tumor, Shanghai Institute of Nutrition and Health, University of Chinese Academy of Sciences, Chinese Academy of Sciences, Shanghai, 200031 China; 4 State Key Laboratory of Medical Genomics, National Research Center for Translational Medicine at Shanghai, Ruijin Hospital Affiliated to Shanghai Jiao Tong University School of Medicine, Shanghai, 200025 China

**Keywords:** Oncolytic virus, PARP1, PD-1, Combination therapy, CRISPR screening

## Abstract

Oncolytic viruses (OVs) offer a novel approach to treat solid tumors; however, their efficacy is frequently suboptimal due to various limiting factors. To address this challenge, we engineered an OV containing targets for neuron-specific *microRNA-124* and *Granulocyte-macrophage colony-stimulating factor* (*GM-CSF*), significantly enhancing its neuronal safety while minimally compromising its replication capacity. Moreover, we identified PARP1 as an HSV-1 replication restriction factor using genome-wide CRISPR screening. In models of glioblastoma (GBM) and triple-negative breast cancer (TNBC), we showed that the combination of OV and a PARP inhibitor (PARPi) exhibited superior efficacy compared to either monotherapy. Additionally, single-cell RNA sequencing (scRNA-seq) revealed that this combination therapy sensitized TNBC to immune checkpoint blockade, and the incorporation of an immune checkpoint inhibitor (ICI) further increased the survival rate of tumor-bearing mice. The combination of PARPi and ICI synergistically enhanced the ability of OV to establish durable tumor-specific immune responses. Our study effectively overcomes the inherent limitations of OV therapy, providing valuable insights for the clinical treatment of TNBC, GBM, and other malignancies.

**Supplementary Information:**

The online version contains supplementary material available at 10.1186/s13045-024-01554-5.

## To the editor

OVs are natural or engineered viruses that selectively replicate within tumors [1]. However, the insufficient replication of OVs inside tumors remains a major obstacle, impeding their efficacy [2–4]. Hence, identifying the molecules that are targetable while replicating restrictive represents a viable strategy [5]. Herein, we developed a neuron-detargeted OV from herpes simplex virus 1 (HSV-1), and found its efficacy-restricting factors in viral replication and immune checkpoint pathways. Additionally, we designed an effective antitumor regimen by precisely combining OV, PARPi, and a programmed cell death protein 1 (PD-1) inhibitor, significantly extending the survival of mice in TNBC, GBM, and melanoma models, potentially supporting direct clinical translation.

**Fig. 1:**
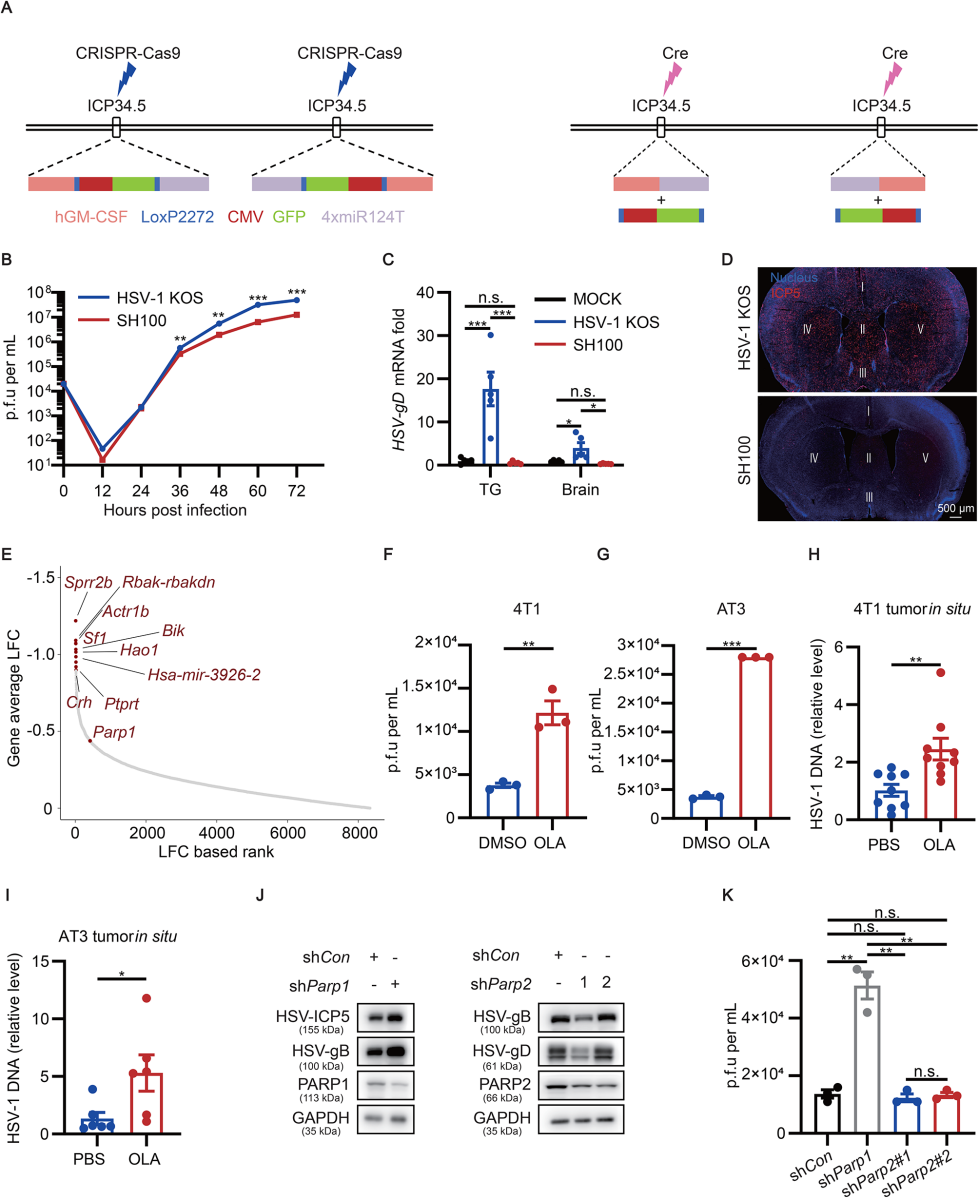
Construction of a neuron-detargeted recombinant oncolytic HSV-1 and identification of its restriction factor PARP1. **A** Schematic illustration for the mechanism of constructing SH100. Donor sequence containing hGM-CSF, GFP and miR124T was inserted in both copies of the *ICP34.5* gene, which was facilitated by CRISPR. The intermediate product SH100-GFP (left) was treated with Cre to remove the GFP cassette to acquire SH100 (right). **B** Comparing replication of HSV-1 KOS and the isolated SH100 strain on Vero cells with one-step growth curve. MOI = 0.05. *n* = 3 per each time point. **C, D** Analysis of SH100 replication in neurons. After cornea infection of HSV-1 KOS and SH100 in the mice (7 dpi in Figs. 1C and 9 dpi in Fig. 1D), viral mRNA was detected by RT-qPCR (**C**), and the virus distribution in the brain was detected by immunofluorescence (**D**). *n* = 5 mice per group. ICP5 was indicated by red, DAPI was indicated by blue. **E** Average MAGeCK analysis for candidate restriction factors from genome-scale CRISPR screening. Top-ranked candidates were labelled. **F**, **G** 4T1 and AT3 cells were treated with 100 µM OLA for 12 h and infected with HSV-1 K26GFP (MOI = 0.8) for an additional 24 h (*n* = 3), followed plaque assay. **H, I** Mice received OLA or PBS intraperitoneal injection (i.p.) for 3 days, followed by intra-tumor injection (i.t.) of SH100 (5 × 10^7^ PFU per mouse). After 2 days, tumors were collected and virus load was detected by qPCR of HSV-1 genomic DNA in 4T1 tumor model (**H**) (*n* = 9 mice per group) and AT3 tumor model (**I**) (*n* = 6 mice per group). **J, K** The impact of different shRNA on HSV replication. 4T1 cell lines were produced by transducing of shRNA-encoding lentiviral vectors and then infected with HSV-1 K26GFP (MOI = 0.8) for 24 h (*n* = 3), followed by Western blot (**J**) and plaque assay (**K**). *P* values were obtained by unpaired two-tailed *t* test (**B, C, F, G, H, I** and **K**). n.s., non-significant; ^*^P < 0.05, ^**^P < 0.01, ^***^P < 0.001. Data presented as the means ± SEM

We engineered SH100 by treating HSV-1 ICP34.5 under the control of *microRNA-124* which specifically expresses in neurons but is often silenced in tumors (Fig. 1A; Additional file 1: Fig. S2A) [6, 7]. One-step growth curves showed that SH100 proliferated as the wild-type virus (HSV-1 KOS) (Fig. 1B). Next, we evaluated the function of GM-CSF inserted in the SH100 and confirmed that SH100 expressed GM-CSF potently in different cell lines (Additional file 1: Fig. S2B-I). Notably, compared to its parent virus, the safety of SH100 greatly improved as indicated by the minimum presence in the trigeminal ganglia (TG) and brain (Fig. 1C, D; Additional file 1: Fig. S3). In contrast, its oncolytic activity was significantly enhanced in various cell types (Additional file 1: Fig. S4 A-H).

Using genome-wide CRISPR screening, we found poly (ADP-ribose) polymerase 1 (*Parp1*) which plays a vital role in DNA repair pathways and NAD^+^ metabolism was among the top candidates (Fig. 1E; Additional file 1: Fig. S5A; Additional file 2: Table S1) [8, 9]. Since PARP1 is the only target with clinically available small molecules, we focused on PARP1 in subsequent studies. We pretreated 4T1 and AT3 cells, respectively, with olaparib (OLA), an inhibitor of PARP1/PARP2, before HSV-1 infection, and found that OLA significantly enhanced the viral replication (Fig. 1F, G; Additional file 1: Fig. S5B-F). Additionally, we confirmed the same observation with additional tumor cell lines in vitro and tumor models in vivo (Fig. 1H, I; Additional file 1: Fig. S5G-L). Extra studies demonstrated that knocking down PARP1 but not PARP2 boosted viral replication (Fig. 1J, K).

**Fig. 2:**
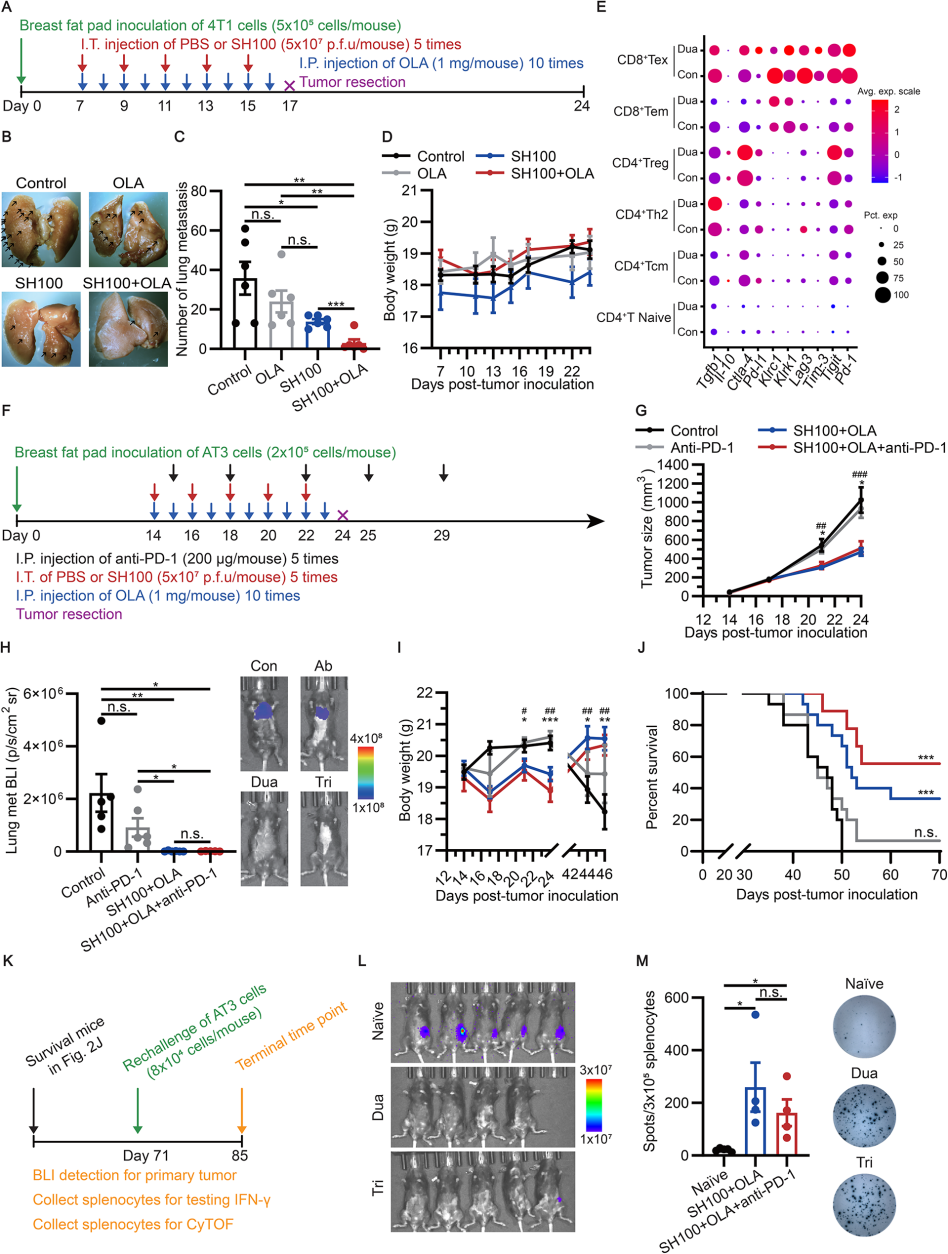
Combination therapy blocked metastasis of TNBC and extended mice survival. **A** Illustration of optimized protocol for dual therapy in 4T1 tumors. **B** Representative images showing lung metastasis. **C** Statistical analysis of the number of lung metastasis. **D** The body weight of BALB/c mice bearing 4T1 tumors upon different treatments (*n* = 6 mice per group). **E** Dot plot showed the expression levels of immune suppressive genes in different subtypes of T cells in the lung metastases of 4T1 models, where dot size and color represent percentage of gene expression (pct. exp) and the averaged scaled expression (avg. exp. scale) value, respectively. **F** Illustration of optimized protocol for triple therapy in AT3 tumors. **G-J** Evaluation the efficacy of optimized triple therapy in AT3 tumors. Tumor growth curves (**G**); lung BLI (**H**) (43 days post tumor inoculation); body weight (**I**); Kaplan-Meier survival curves of C57BL/6J mice bearing AT3 tumors (**J**). (**G, I** and **J**) Control, *n* = 15, Anti-PD-1, *n* = 15, SH100 + OLA, *n* = 15, SH100 + OLA + anti-PD-1, *n* = 9. (**H**) Control, *n* = 5, Anti-PD-1, *n* = 6, SH100 + OLA, *n* = 6, SH100 + OLA + anti-PD-1, *n* = 6. In (**G** and **I**), the symbol “^*^” denotes the difference between Control and SH100 + OLA + anti-PD-1. The symbol “^#^” denotes the difference between Control and SH100 + OLA. **K** Rechallenge scheme for survived mice in AT3 tumors. **L** Whole-body BLI analysis of tumor burden 14 days after AT3 cells reinoculation. **M** Interferon-γ (IFN-γ) ELISPOT analysis of splenocytes harvested 14 days after AT3 reinoculation. *P* values were obtained by unpaired two-tailed *t* test (**C, G, H, I** and **M**), n.s., non-significant, ^*, #^*P* < 0.05; ^**, ##^*P* < 0.01; ^***, ###^*P* < 0.001, or Mantel-Cox test (**J**), n.s., non-significant, ^***^P < 0.001. Data were shown as the means ± SEM

Next, we evaluated the synergistic antitumor effect of SH100 and OLA using a 4T1 TNBC lung metastasis model (Fig. 2A). Remarkably, the dual therapy exhibited a significantly reduced lung metastasis compared to SH100 alone, without affecting the body weight of the mice (Fig. 2B-D). We also demonstrated this synergistic effect in a GL261n-1 GBM model (Additional file 1: Fig. S6A-C). In addition, we examined the innate immune responses within primary tumors and found that intra-tumor injection of SH100 triggered the innate immune sensing, without detecting a significant difference to the SH100 + OLA group. (Additional file 1: Fig. S6D-I).

We then used scRNA-seq to analyze the status of T cells in 4T1 lung metastases models (Additional file 1: Fig. S7A-D). We found multiple immune checkpoint genes were upregulated in CD4^+^Treg after dual therapy (Fig. 2E). Therefore, we supplemented the dual therapy with a PD-1 inhibitor. Indeed, we found triple therapy could further reduce lung metastases compared to dual therapy (Additional file 1: Fig. S8A, B). Additionally, we found increased lymphocyte infiltration, increased CD8^+^ PD-1^+^ T cells, and upregulation of immunosuppressive genes associated with M2-like macrophages in primary tumors (Additional file 1: Fig. S8, S9). As lung metastasis was still detected in nearly all mice, we reasoned it was due to delayed PD-1 antibody administration. Therefore, we optimized the regimen by injecting PD-1 antibodies only one day instead of five days after SH100 administration and evaluated efficacy in 4T1 and AT3 TNBC models, respectively (Fig. 2F; Additional file 1: Fig. S10A) [10]. The dual and triple regimens significantly suppressed primary tumor growth and alleviated lung metastasis in the AT3 model without significant toxicity (Fig. 2G-I; Additional file 1: Fig. S10B, C). However, the triple therapy showed the highest survival rates in both 4T1 and AT3 models, compared to dual therapy and the PD-1 inhibitor (Fig. 2J; Additional file 1: Fig. S10D), which was also validated in the B16F10n-1 melanoma model (Additional file 1: Fig. S10E-H).

To investigate tumor-specific immunological memory, we performed rechallenge study and found nearly all mice in combination groups were tumor-free except one with weak signals (Fig. 2K, L). IFN-γ enzyme-linked immunospot (ELISPOT) and cytometry by time of flight (CyTOF) analysis indicated that the combination therapy established long-term and systematic tumor-specific immunological memory (Fig. 2M; Additional file 1: Fig. S11-S12).

In summary, we developed a microRNA-regulated OV and found a triple combination therapy that efficiently overcame multiple constraints and significantly enhanced the antitumor effects. Our study may enhance the clinical efficacy of oncolytic therapy, providing better clinical translation for cancer patients.

### Electronic supplementary material

Below is the link to the electronic supplementary material.

Supplementary Material 1

Supplementary Material 2

Supplementary Material 3

Supplementary Material 4

Supplementary Material 5

Supplementary Material 6

## Data Availability

Single-cell RNA sequencing and Bulk RNA-sequencing datasets generated in this study are available on the GEO database under the accession numbers GSE207977 and GSE201760, respectively. Materials generated in this study are available upon request.

## Abbreviations

OV: Oncolytic virus
GM-CSF: Granulocyte-macrophage colony-stimulating factor
GBM: Glioblastoma
TNBC: Triple-negative breast cancer
PARPi: PARP inhibitor
scRNA-seq: Single-cell RNA sequencing
ICI: Immune checkpoint inhibitor
HSV-1: Herpes simplex virus 1
PD-1: Programmed cell death protein 1
TG: Trigeminal ganglia
PARP1: Poly (ADP-ribose) polymerase 1
OLA: Olaparib
ELISPOT: Enzyme-linked immunospot
CyTOF: Cytometry by time of flight
