# Supplementary material for: Identification of restrictive molecules involved in oncolytic virotherapy using genome-wide CRISPR screening
Source: J Hematol Oncol. 2024 May 23;17:36. doi: 10.1186/s13045-024-01554-5 (PMC11118103; doi:10.1186/s13045-024-01554-5)
Supplement: Supplementary file 1 — Supplementary Material 1 [file 13045_2024_1554_MOESM1_ESM.docx]

**Supplemental Methods**

**Cell culture**

HEK293T, HEK293, Vero, MDA-MB-231, MDA-MB-468, B16F10, U251, U87, A375, AT3, GL261, MC38, and HEB cells were maintained in Dulbecco’s modified Eagle’s medium (DMEM) (Gibco). 4T1, THP-1, and THP-1 KO-STING cells were maintained in RPMI 1640 (Gibco). DMEM and RPMI 1640 were supplemented with 10% fetal bovine serum (FBS) (Gibco) and 1% penicillin/streptomycin (P/S) (Thermo Fisher). All cells were cultured at 37 °C under 5% (v/v) CO_2_.

**Viruses**

The wild-type HSV-1 strain KOS and its derivatives, HSV-1 K26GFP and HSV1-GFP, were utilized in this study, and these viruses were obtained from Soren Riis Paludan at Aarhus University, Denmark. Specifically, the construction of HSV-1 K26GFP was achieved by homologous recombination, in which the GFP open reading frame was placed at the 13th position after the start codon of the UL35 gene of HSV-1 KOS, so that GFP could be transcribed under the control of the UL35 promoter and fused with the capsid protein VP26 [1]. This allowed the produced viral particles to enclose GFP proteins. Additionally, the construction of HSV1-GFP was achieved by inserting the CMV-GFP into the UL49.5 gene of the HSV-1 KOS. In HSV1-GFP, the virus particles have GFP gene, but do not have GFP proteins. [2]. Only after the virus infects the cell can the GFP protein be expressed, which allows for the visually observation of positive cells infected by the virus, and the assessment of the generation of progeny virus particles based on the strength of GFP expression. The schematic diagram of the structure of the aforementioned virus is provided in Additional file 1: Fig. S1.

**Mouse nectin-1 stable transduction**

To investigate the efficacy of SH100 in melanoma and GBM, we generated modified B16F10 melanoma and GL261 GBM models, respectively. Due to the lack of nectin-1 (the key HSV-1 receptor) expression, original B16F10 and GL261 cells are difficult to be infected by HSV-1 [3, 4]. To increase the permissiveness, we used lentiviral vectors to transduce *mouse nectin-1* into B16F10 and GL261 cells, thus enabling the stable expression of the mouse nectin-1 protein. The nectin-1 inserted B16F10 and GL261 cells were named B16F10n-1 and GL261n-1, respectively. Specifically, the lentiviral vector was produced by transfecting of HEK293T cells with pCDH-CMV-mNectin-1-EF1α-Puro, pMD2.G and pSPAX. B16F10 or GL261 cells were seeded into 6-well plates at a density of 1 × 10^5^ cells/well. The next day, the cells were infected with 100 ng of lentiviral vector. Three days later, 2 µg/mL puromycin (Beyotime, ST551) was added to enrich the positive cells.

**shRNA-mediated silencing**

The lentiviral shRNA expression plasmid pGPU6/GFP/Neo (Clontech) was used to generate stable gene expression knockdown in 4T1 cells. The targeting sequences used in this study are listed in Additional file 2: Table S3. The lentiviral vector was produced by transfecting of HEK293T cells with the shRNA vector, pMD2.G and pSPAX. 4T1 cells were seeded into 6-well plates at a density of 1 × 10^5^ cells/well. The next day, 4T1 cells were infected with 100 ng of lentiviral vector. Three days later, 2 µg/mL puromycin (Beyotime, ST551) was added to enrich the positive cells.

**Generation of recombinant oncolytic HSV-1**

The donor plasmid comprising GM-CSF, CMV-GFP, miR124T and the homologous arms of *ICP34.5* was obtained by multi-fragment homologous recombination. Specifically, the *GM-CSF* gene, *CMV-GFP* cassette (with Loxp2272 sites) and a miR124T box were inserted downstream of *ICP34.5* (in both copies of the *ICP34.5* gene) via CRISPR-boosted homologous recombination. The lenti-CRISPR-v2 plasmid targeting *ICP34.5* was constructed by using T4 DNA ligase, and the sgRNA sequence was CGGAGCCGGCCCGGCGAACT. HEK293T cells were seeded into a 6-well plate at a density of 6 × 10^5^/well 24 h before co-transfection of the donor plasmid and lenti-CRISPR-ICP34.5. One day after transfection, 1 µg/mL puromycin was added to screen for the positive cells. Forty-eight hours later, the HEK293T cells were infected with HSV-1 KOS at an MOI of 1.0 for 36 h before the supernatants were collected for plaque formation assays to purify the recombinant HSV-1 with GFP. For plaque assay purification, Vero cells were seeded into 6-well plates, followed by growth to a confluent monolayer. Subsequently, the supernatant from the HEK293T cells was subjected to gradient dilution before being added to the cell culture medium. After 2 h, the supernatant was discarded, and 1% agarose supplemented with 1 × DMEM and 2% FBS was added. After 72 h, the recombinant HSV-1 with GFP was observed and removed via fluorescence microscopy. For deletion of the *CMV-GFP* cassette, HEK293T cells were seeded into a 6-well plate at a density of 6 × 10^5^/well 24 h before transfection of pCCL-PGK-Cre. Twenty-four hours later, the HEK293T cells were infected with recombinant HSV-1 with GFP at an MOI of 1.0 for 36 h before the supernatants were collected for plaque formation assays to purify the recombinant HSV-1 without GFP (SH100). The steps of plate assay purification experiment were the same as those mentioned above.

**Virus production and plaque assay**

The HSV-1 strains used in this study included HSV-1 KOS, HSV-1 K26GFP (the HSV-1 KOS strain with GFP in its capsid), and HSV1-GFP (the HSV-1 KOS strain expressing GFP driven by the CMV promoter). The production and purification of HSV-1 were performed on Vero cells. Viral titers were determined by plaque assay. In brief, the virus supernatant was gradient diluted and added to a monolayer of Vero cells. Two hours later, the medium was replaced with DMEM supplemented with 1% agarose (Sangon, A600015) and 2% FBS. Plaques were counted 72 h later. To compare the relative levels of infection and replication of HSV-1 KOS and SH100 in different cell types, we performed a quantitative plaque size analysis on normal human glial cells, glioma cells, melanoma cells, and breast cancer cells at 48 h post-infection. Monolayer cells were infected at an MOI of 0.01. Two hours later, the inoculum was removed, and the cells were washed twice with PBS before the addition of 1% CMC in MEM, which was used as an overlay. Two days later, the plaque density was determined via crystal violet staining. The plaque size was measured (*n*=60 plaques/cell type/virus), and the means and standard errors of the means (SEMs) were used to compare the infection and replication of the two viruses.

**Genome-wide CRISPR screening**

HEK293 cells were infected with lenti-Cas9, followed by 16 μg/mL blasticidin selection for 72 h. After selection, single cells were seeded into 96-well plates to obtain a cell line stably expressing Cas9. The human CRISPR knockout pooled library (GeCKO v2) was acquired from Addgene (#1000000049). A total of 1.63×10^8^ Cas9-expressing HEK293 cells were seeded into 6-well plates (16 wells) and infected with human CRISPR knockout pooled library A at an MOI of 0.3 and selected using 2 μg/mL puromycin for 72 h. For screening, 6.5×10^7^ cells (genome knockout pool and control cells) were seeded (at a coverage of 1000×) and then infected with HSV-1 KOS at an MOI of 1, and parallel screening was performed. The cells were collected for genomic DNA extraction and sequencing 4 days after infection. Uninfected cells were used as controls. The raw FASTQs were aligned to the library and processed into counts for each sgRNA using the MAGeCK count command with automatic trimming of the 5’ end of the reads in MAGeCK software (MAGeCK enables robust identification of essential genes from genome-scale CRISPR/Cas9 knockout screens), and the MAGeCK RRA command was used for calculating the log2-fold change in all genes independently for each comparison. Nontargeting sgRNAs were used to normalize the sequencing reads and generate a null distribution to determine *P*-values.

**Mice**

Female BALB/c and C57BL/6J mice aged 6 to 8 weeks were obtained from Beijing Vital River Laboratory Animal Technology Co., Ltd. The animals were housed in a specific pathogen-free environment under a 12 h light/12 h dark cycle. All animal-related experiments were performed under the guidelines of the Institutional Animal Care and Use Committee (IACUC) of Shanghai Jiao Tong University with approval from the animal ethics committee.

***In vivo* experiments and tumor models**

For the safety test, C57BL/6J mice were infected with 2 × 10^6^ PFU of HSV-1 KOS or SH100 in the eyes. Seven days after infection (Fig. 1C) or 9 days after infection (Fig. 1D, Fig. S3), the mice were euthanized, and TG and brain samples were collected, and subjected to RT‒qPCR or immunofluorescence.

For the evaluation of innate immune response within primary tumors, 5 × 10^5^ cells/100 µL of 4T1 cells or AT3 cells were injected into the second mammary fat pad on the right side of BALB/c mice or C57BL/6J mice. When tumors reached 50-100 mm^3^, mice were randomly grouped. 1 mg OLA (Energy Chemical, E0803880010) in a volume of 100 µL was injected intraperitoneally once per day. Three days later, SH100 (5 × 10^7^ PFU in 100 µL) were injected intratumorally. After 24 hours, the tumors were harvested and subjected to RT-qPCR analysis. The primers used are detailed in Additional file 2: Table S3.

For the viral replication experiments *in vivo*, 5 × 10^5^ cells/100 µL of 4T1 or AT3 cells were injected into the second mammary fat pad on the right side of BALB/c or C57BL/6J mice, respectively; 5 × 10^5^ B16F10n-1 cells were injected subcutaneously into the back of C57BL/6J mice. When tumors reached 50-100 mm^3^, mice were randomly grouped. 1 mg OLA (Energy Chemical, E0803880010) in a volume of 100 µL was injected intraperitoneally once per day. Three days later, SH100 (5 × 10^7^ PFU in 100 µL) were injected intratumorally. After 2 days, tumors were harvested and minced in 1 mL PBS. Samples were subject to 3 freeze-thaw cycles followed by a centrifugation at 12,000 g for 5 min, and 200 µL supernatant was used to extract the HSV-1 genomic DNA using Viral RNA/DNA Extraction Kit (Takara, 9766). Viral load was quantified by qPCR, and the primers used are listed in Additional file 2: Table S3.

For the GL261n-1 tumor model, 3 µL of 1 × 10^4^ GL261n-1 cells were injected into the right hemisphere (1.5 mm right and 1.5 mm down to bregma at a 3 mm depth) of C57BL/6J mice. Three days later, SH100 (1 × 10^6^ PFU in 3 µL) was injected into the same site. One mg OLA in a volume of 100 µL was injected intraperitoneally once per day from Day 3 to 5 for 3 injections.

For the 4T1 tumor model, 4T1 cells in the logarithmic growth phase were digested and prepared as a cell suspension at a concentration of 5 × 10^5^ cells/100 µL, which was subsequently injected into the second mammary fat pad on the right side of BALB/c mice. When 4T1 tumors reached 50-100mm^3^, the mice were randomly grouped. For the AT3 tumor model, AT3 cells were implanted into C57BL/6J mice at a concentration of 2 × 10^5^ cells/100 µL. Two weeks later, the mice were randomly grouped. For treatments, SH100 (5 × 10^7^ PFU) in a 100 µL volume was injected intratumorally every other day from Day 7 to 15 (4T1 mice model) or from Day 14 to 22 (AT3 model) for 5 injections. OLA (1 mg in a volume of 100 µL) was injected intraperitoneally once per day from Day 7 to 16 (4T1 model) or from Day 14 to 23 (AT3 model) for 10 injections. Anti-PD-1 (BioXCell, clone RMPI-14) was injected intraperitoneally at a dose of 200 µg on Days 12, 15, and 19; on Days 6, 9, 13, 17, and 20 for the 4T1 model; or on Days 15, 18, 22, 25, and 29 for the AT3 model for 5 injections. One day after the last injection of OLA, the primary tumor was removed surgically. For Fig. 2B and S8B, the mice were euthanized 7 days after surgical removal of the primary tumor, and the number of lung metastases was calculated.

For the AT3 rechallenge study, 8 × 10^4^ cells were injected into the second mammary fat pad on the left flank of C57BL/6J mice. Tumor sizes were recorded 14 days after AT3 rechallenge by bioluminescence imaging (BLI). Mice were euthanized and the spleens were collected to perform ELISPOT.

For the B16F10n-1 model, 5 × 10^5^ B16F10n-1 cells were injected subcutaneously into the backs of C57BL/6J mice. When the B16F10n-1 tumors reached approximately 50 mm^3^, the mice were randomly grouped. For treatments, SH100 (5 × 10^7^ PFU) in a 100 µL volume was injected intratumorally every other day from Day 5 to 13 for 5 injections. OLA (1 mg in a volume of 100 µL) was injected intraperitoneally once per day from Days 5 to 14 for 10 injections. Anti-PD-1 was injected intraperitoneally at a dose of 200 µg on Days 6, 9, and 13 for 3 injections.

***In vivo* bioluminescence imaging**

For the AT3 tumor model, mice were injected intraperitoneally with 200 µL of D-luciferin (15 mg/mL) (Yeasen, 40902ES03). Then, the mice were anesthetized in a chamber with 30% isoflurane. Approximately 10 min after the injection of D-luciferin, bioluminescence imaging of luciferase expression was performed with an IVIS system (PerkinElmer). The lung and whole-body BLI were quantified as average radiance.

**Enzyme-linked immunospot (ELISPOT)**

Splenocytes were harvested 14 days after tumor cell rechallenge, and a mouse IFN-γ ELISPOT Kit (Mabtech, 3321-4HST-2) was used to detect specific T-cell responses to tumor cells. There were 3×10^5^ splenocytes in each well. ELISPOT data were collected with an S6 FluoroCore immunospot.

**Enzyme linked immunosorbent assay (ELISA)**

Twenty-four hours after virus infection, the cell culture supernatants were collected and an ELISA kit (Solarbio, SEKH-0057) was used to detect the GM-CSF content in the supernatant, in accordance with the instructions outlined in the manual. The optical density (OD) at 450 nm was collected using Epoch (BioTek).

**Single-cell RNA sequencing**

A single-cell RNA sequencing experiment was performed by NovelBio Bio-Pharm Technology Co., Ltd. The scRNA-Seq libraries were generated using the 10×Genomics Chromium Controller Instrument and Chromium Single Cell 5’ V1.1 Reagent Kits (10×Genomics, Pleasanton, CA). Briefly, cells were concentrated to 1,000 cells/µL and approximately 10,000 cells were loaded into each channel to generate single-cell Gel Bead-In-Emulsions (GEMs), which resulted in the expected mRNA barcoding of 6,000 single cells for each sample. After the RT step, GEMs were broken and barcoded cDNA was purified and amplified. The amplified barcoded cDNA was fragmented, A-tailed, ligated with adaptors and index PCR-amplified. The final libraries were quantified using the Qubit High Sensitivity DNA assay (ThermoFisher) and the size distribution of the libraries was determined using a High Sensitivity DNA chip on a Bioanalyzer 2200 (Agilent). All libraries were sequenced by Illumina Novaseq 6000 (Illumina) on a 150-bp paired-end run.

**Flow cytometry**

To isolate tumor cells, tumors were finely minced and digested with an enzyme mixture [1 mg/mL collagenase (Sigma, 10269638001), 0.1 mg/ml hyaluronidase (Sigma, H3506) and 125 U/ml DNase I type IV (Sigma, D5025)] at 37 ℃ for 90 min. For splenocyte extraction, spleens were collected and ground gently in PBS. The mixture of tumor cells or splenocytes was harvested and pushed through a 70-µm strainer (BD Falcon, 352350) before RBC lysis (Sangon, B541001). The cells were washed and resuspended in PBS (Gibco). Next, the cells were stained with various combinations of the indicated antibodies for 30 min on ice in the dark, after which flow cytometry (BD LSRFortessa, BD Biosciences) was used for analysis. All antibodies used in this study are provided in Additional file 2: Table S4. For the detection of GFP^+^ cells, as shown in Additional file 1: Fig. S5B, cells were harvested with 0.05% trypsin (Thermo Fisher), washed with PBS, and then fixed with 4% PFA for 20 min at RT. Later, the cells were washed with PBS and subjected to flow cytometry analysis. Data analysis and compensation were performed with BD FACSDiva 7 and FlowJo 7.6. The gating scheme was presented in Additional file 1: Fig. S13A.

**Histological analysis**

Tumors were harvested and fixed in 4% PFA (Servicebio, G1101) in PBS overnight. The tumors were then embedded in paraffin and cut into sections. The sections were stained with specific antibodies after rehydration through a graded series of alcohol and citrate buffer (10 mM sodium citrate, pH 6.0). All the antibodies used are listed in Additional file 2: Table S4. The sections were scanned by a Pannoramic 250FLASH (3DHISTECH).

**Bulk RNA-seq analysis**

Total RNAs from primary tumors (Control, SH100+OLA, SH100+OLA+anti-PD-1) were extracted by TRIzol (ThermoFisher). RNA integrity was assessed using the RNA Nano 6000 Assay Kit on the Agilent Bioanalyzer 2100 system (Agilent). Sequencing libraries were generated using NEBNext Ultra^TM^ RNA Library Prep Kit for Illumina (NEB) following manufacturer’s recommendations and index codes were added to attribute sequences to each sample. The clustering of the index-coded samples was performed on a cBot Cluster Generation System using TruSeq PE Cluster Kit v4-cBot-HS (Illumina) according to the manufacturer’s instructions. After cluster generation, the library preparations were sequenced on an Illumina platform and paired-end reads were generated. The raw reads were further processed with the bioinformatics pipeline tool BMKCloud (www.biocloud.net) online platform.

**Quantitative PCR**

Total RNA was extracted using an RNA isolate (Vazyme, R401-01) before cDNA was generated using HiScript III qRT SuperMix (Vazyme, R323-01). qPCR was performed using ChamQ Universal SYBR qPCR Master Mix (Vazyme, Q711-02). The sequences of primers used are listed in Additional file 2: Table S3. All qPCR experiments were performed using a real-time PCR system (LightCycler 96, Roche).

**Western blot analysis**

Cell lysates were generated using Cell Lysis Buffer (Beyotime, P0013). Protein samples were separated using 12% bis-tris protein gels and transferred to polyvinylidene difluoride (PVDF) membranes (GE Healthcare). The membranes were blocked with 5% fat-free milk dissolved in PBS for 2 h at RT, and incubated overnight at 4 ℃ with diluted primary antibodies in primary antibody dilution buffer (Beyotime, P0256). After incubation with secondary antibodies, the membranes were visualized using gel imaging system (Amersham ImageQuant 680, GE). All antibodies used are listed in Additional file 2: Table S4.

**Nuclear and cytoplasmic fractionation**

Cells in the indicated groups were harvested in 0.05% trypsin and washed with PBS. The cells were lysed using 0.1% NP40 lysis buffer (Beyotime, P0013F) diluted in PBS and centrifuged at 4 ℃, 1000 g × 5 min. The cytoplasmic and nuclear fractions were separated from the supernatants and precipitated, respectively. Finally, the whole-cell lysates and cytoplasmic and nuclear fractions were analyzed via Western blotting.

**Immunofluorescence**

Cells in 24-well plates were fixed with 4% PFA for 20 min at RT, and permeabilized with 70% ethyl alcohol (Aladdin, E111991) for 20 min at -20 ℃. Cell nuclei were stained with Antifade Mounting Medium with DAPI (Beyotime, P0131).

For the safety tests of SH100 *in vivo* (Fig. 1D and Fig. S3), the brain was fixed with 4% PFA at 4 ℃ for 24 hours, then subjected to a dehydration treatment in a 30% sucrose solution at 4°C for 48 hours. The samples were placed in an embedding capsule, which were subjected to OCT embedding. The embedding capsule was subsequently placed in a -80°C freezer, allowing the sample to fully solidify. The sample was subjected to frozen sectioning, with a thickness of 15 µm, and the slices were placed in a -80°C freezer for storage. Next, the slices were allowed to air-dry at room temperature for 10 min, and outlined using a PAP pen. Then, the procedure involved with the steps of blocking (2% goat serum), primary antibody (anti-HSV1/2 ICP5) incubation, and a subsequent secondary antibody (Alexa Fluor 555 Conjugated Goat anti-mouse IgG Goat Polyclonal Antibody) incubation. The cell nuclei were stained with Antifade Mounting Medium with DAPI. The antibodies ultilized are listed in Additional file 2: Table S4.

Images were acquired by using laser scanning confocal microscope (A1si, Nikon) or fluorescence microscope (Pannoramic DESK, P-MIDI, P250, 3D HISTECH) with Pannoramic Scanner software.

**CyTOF analysis of all immune cells in the spleen**

The immune cells of the spleen were detected using CyTOF analysis. Single cell suspensions (3×10^6^ cells) of the spleen were collected. Cells were washed once with 1×PBS and then stained with 100 μL of 250 nM cisplatin (Fluidigm) for 5 min on ice to exclude dead cells, and then incubated in Fc receptor blocking solution before stained with surface antibodies cocktail for 30 min on ice. For intracellular staining, cells were washed twice with FACS buffer (1×PBS+0.5%BSA) and fixed in 200 μL of intercalation solution (Maxpar Fix and Perm Buffer containing 250 nM 191/193Ir, Fluidigm) overnight. After fixation, cells were washed once with FACS buffer and then perm buffer (eBioscience), stained with intracellular antibodies cocktail for 30 min on ice. Cells were washed and resuspended with deionized water, added into 20% EQ beads (Fluidigm), acquired on a mass cytometer (Helios, Fluidigm). Data of each sample were debarcoded from raw data using a doublet-filtering scheme with unique mass-tagged barcodes. Each fcs file generated from different batches was normalized through the bead normalization method. CD45^+^ cells were manually gated using FlowJo software to exclude debris, dead cells and doublets, leaving live, single immune cells. Apply the PARC clustering algorithm to all cells to partition the cells into distinct phenotypes based on marker expression levels. Annotate cell type of each cluster according to its marker expression pattern on a heatmap of cluster vs marker. Use the dimensionality reduction algorithm t-SNE to visualize the high-dimensional data in two dimensions and show the distribution of each cluster and marker expression and difference among each group or different sample types. The relative panels were listed in Additional file 2: Table S5 and the gating scheme was presented in Additional file 1: Fig. S13B.

**Statistical analysis**

All statistical analyses were performed using GraphPad Prism 8.0. Two-tailed unpaired Student’s *t*-test was used in this study. Survival curves were analyzed using the Mantel-Cox test. Data are presented as the means ± SEM., and *P* values are indicated by ^*^*P*<0.05; ^**^*P*<0.01; ^***^*P*<0.001.

**Supplemental Results**

**Construction of a neuron-detargeted recombinant oncolytic HSV-1**

Natural HSV-1 is a neuron-tropic virus that must be engineered for oncolytic purposes. As ICP34.5 is a neurovirulence factor [5], it was deleted in early generations of oncolytic HSV infection [6, 7]. However, ICP34.5 also plays an important role in fighting the host antiviral response and promoting viral replication. In this study, we engineered an oncolytic HSV-1 strain (designated SH100) by treating *ICP34.5* under the control of *microRNA-124*, which is specifically expressed in neurons but often silenced in tumors [8, 9], instead of simply deleting *ICP34.5* (Fig. 1A; Additional file 1: Fig. S2A). Upon entering neuronal cells, *microRNA-124* binds to its target sequence located in the 3' UTR region of the *ICP34.5* mRNA, causing the mRNA to degrade and preventing ICP34.5 from being expressed. Therefore, the replication of SH100 in neuronal cells was inhibited. To determine the replication kinetics of SH100, we generated one-step growth curves on Vero cells. As presented in Fig. 1B, the miRNA-regulated SH100 strain exhibited replication kinetics similar to those of the wild-type strain (HSV-1 KOS) at early time points but was slightly reduced at late time points. To assess the successful construction of SH100, we infected a variety of cell lines with SH100 or HSV-1 KOS, and evaluated the expression of GM-CSF at both the mRNA level in the cells and the secreted protein level in the culture supernatant. The results showed that SH100 was capable of expressing a substantial quantity of GM-CSF in comparison to the parental strain HSV-1 KOS (Additional file 1: Fig. S2B-I). Notably, compared with the presence of the parent virus, the safety of SH100 greatly improved, as indicated by the minimum presence of SH100 in the trigeminal ganglia (TG) and brain (Fig. 1C, D; Additional file 1: Fig. S3).

Next, we verified the oncolytic activity of SH100 and HSV-1 KOS in different cancer cell types. We found that the oncolytic activity of SH100 was significantly greater than that of the original HSV-1 KOS in a variety of cell lines, including MDA-MB-231, MDA-MB-468, B16F10n-1, U251, U87, and A375 cells, and was comparable in HEB cells (normal human glial cell line) and 4T1 cells (Additional file 1: Fig. S4 A-H), as indicated by the plaque size. Taken together, these results indicate that SH100 has an improved safety profile and oncolytic activity comparable, if not better, than that of the original HSV-1 KOS.

**Genome-wide CRISPR screening identified PARP1 as an intrinsic OV restriction factor**

To determine the intrinsic restrictive factors for optimizing OV performance, we conducted genome-wide CRISPR screening. We first generated Cas9-expressing HEK293 cells and then infected the cells with a CRISPR library at an MOI of 0.3, followed by puromycin selection to remove uninfected cells. Next, we challenged CRISPR library-containing cells with HSV-1 (KOS strain) to test the hypothesis that knockout of restrictive factors would facilitate cell death and reduce the abundance of the corresponding gRNAs. The remaining cells were harvested for deep sequencing and bioinformatic analysis after 4 days of culture (Additional file 1: Fig. S5A and Methods). We found poly (ADP-ribose) polymerase 1 (*Parp1*) which plays an important role in various DNA repair pathways, and that NAD^+^ metabolism was among the top candidate restriction factors [10, 11] (Fig. 1E and Additional file 2: Table S1). As PARP1 is the only target of clinically available small molecules for regulation [12], we focused on PARP1 in subsequent studies.

To confirm the antiviral function of PARP1, we pretreated 4T1 cells (a mouse breast cancer cell line) with olaparib (OLA), an inhibitor of PARP1/PARP2 [30], before infection with HSV-1. We found that PARP inhibition significantly increased HSV1-GFP infection, as shown by the increased percentage of GFP^+^ cells (Additional file 1: Fig. S5B) and increased transcription of the viral gD gene (Additional file 1: Fig. S5C). Additionally, the increase in infection density was also confirmed by confocal microscopy analysis of the HSV-1 K26GFP infection (Additional file 1: Fig. S5D). We found that more GFP-labeled viral capsids were detected in the nucleus, cytoplasm and cell membranes in the OLA-treated group than in the non-OLA group, suggesting that OLA treatment facilitated viral replication (Additional file 1: Fig. S5D). Next, we analyzed the expression levels of viral gB and gD in the nucleus, cytoplasm and whole cells, and found more HSV-1 gB and gD proteins in the OLA group than in the control group for all the other cell parts (Additional file 1: Fig. S5E, F). Moreover, OLA treatment significantly enhanced the number of HSV-1 progeny in the infected 4T1 cells, as indicated by the plaque-forming units (PFUs) in the supernatants of the infected cells (Fig. 1F). Additionally, we confirmed the same observation with other tumor cell types, including AT3, B16F10n-1 GL261n-1, and HeLa cells (Fig. 1G; Additional file 1: Fig. S5G-I). Interestingly, we found that OLA cotreatment reduced progeny production in THP-1 cells (Additional file 1: Fig. S5J), but these effects could be reversed when STING was knocked out (Additional file 1: Fig. S5K). To further elucidate the impact of PARP1 on viral replication, we performed intratumor viral replication experiments using two TNBC models and one melanoma model (Fig. 1H, I and Additional file 1: Fig. S5L). The results demonstrated a significant increase in viral load within tumors receiving the combined treatment of OLA (Fig. 1H, I and Additional file 1: Fig. S5L).

Given that OLA can target both PARP1 and PARP2, we attempted to knock out *Parp1* or *Parp2* via CRISPR but failed to obtain double-allele knockout clones, suggesting that residual expression of PARP is necessary for normal function. We therefore designed *Parp1* and *Parp2* shRNAs instead and determined their impact on virus replication. We found that shRNA-*Parp1* and shRNA-*Parp2* could effectively knock down the protein expression of PARP1 and PARP2 in 4T1 cells, as shown by Western blot analysis (Fig. 1J). Notably, we found that knocking down PARP1 but not PARP2 boosted the expression of the two viral glycoproteins or the production of progeny virus after HSV-1 K26GFP infection (Fig. 1J, K). Overall, we identified PARP1 as an intrinsic restriction factor during HSV-1 replication and found that blocking PARP1 significantly enhanced HSV-1 replication *in vitro* and *in vivo*.

**A PARP inhibitor improved OV treatment outcomes in GL261n-1 GBM model and 4T1 TNBC model**

To investigate whether PARP1 inhibition could improve the efficacy of oncolytic virus *in vivo*, we compared oncolytic monotherapy and combination therapy with a PARP1 inhibitor in a GL261n-1 glioblastoma multiforme (GBM) model (Additional file 1: Fig. S6A). Although intratumor injection of the virus resulted in slight weight loss, the mice returned to a normal weight after 7 days (Additional file 1: Fig. S6B). Notably, the survival of the dual-treated mice was significantly prolonged, which was not achieved by SH100 or OLA alone (Additional file 1: Fig. S6C). The poor prognosis of TNBC patients is associated with multiorgan metastasis, among which lung metastasis is one of the most common distant metastases [13]. To test whether OV administration could diminish primary tumor size and reduce tumor metastasis, we constructed a TNBC lung metastasis model by injecting of 4T1 cells into the right fat pad of mice and then removing the primary tumor after OV treatment to more closely mimic clinical practice, as surgery is a key component of current breast cancer treatments (Fig. 2A). We found that lung metastasis was significantly inhibited by SH100 but not by OLA alone, whereas their combination significantly outperformed OV monotherapy (Fig. 2B, C). Additionally, no significant difference in body weight was observed among the different treatment groups, suggesting that high-dose dual therapy was tolerated by the mice (Fig. 2D). Studies have shown that PARP1 plays a role in the innate immune response mediated by HSV-1 [14]. Therefore, we sought to assess the expression levels of several innate immune response related genes within primary tumors (4T1 and AT3), including interferon beta 1 (*Ifnb1*), IFN-stimulated genes 15 (*Isg-15*), and small inducible cytokine subfamily B member 10 (*Cxcl10*). The results revealed that the intra-tumoral injection of SH100 could significantly upregulate the expression of innate immune response products within primary tumors, with no significant difference between the SH100+OLA group (Additional file 1: Fig. S6D-I). The results suggested that the innate immune response within primary tumors was mainly triggered by viruses and may not be significantly associated with PARP1. These findings suggest that OLA can augment the therapeutic efficacy of SH100 *in vivo*, exhibiting a pronounced synergistic effect.

**Dual therapy with OV and Olaparib sensitizes 4T1 tumors to immune checkpoint inhibitor**

To further understand the changes in the immune status of T cells in the 4T1 model after OV and OLA treatment, we collected lung metastatic tumor samples from nontreated controls and from SH100 and OLA cotreated mice for scRNA-seq (Additional file 1: Fig. S7A, B) and Methods). To investigate the changes in T cell immune status before and after treatment, we conducted a comparative analysis of all differentially expressed genes (DEGs) in T cells between the control group and the dual therapy group. Interestingly, we found that the expression of multiple immune suppressive genes, including *Lag3*, *Hilpda*, *Pd-1*, *Ccr8*, and *S100a4*, decreased in the dual therapy group compared to the non-treated control, suggesting that our oncolytic dual therapy may enhance the T cell function (Additional file 1: Fig. S7C). Subsequently, T cells were subtyped into CD4^+^ Tcm, CD4^+^ Th2, CD4^+^ Naïve, CD4^+^ Treg, CD8^+^ Tex, and CD8^+^ Tem cells based on the expression of marker genes (Additional file 1: Fig. S7D), and the expression of several immune-suppressive genes on various subtypes of T cells were analyzed. Notably, the results revealed a tendency toward upregulation of several immune checkpoint genes, including *Pd-1*, *Pd-l1*, *Ctla-4*, and *Tigit*, in CD4^+^ Tregs (Fig. 2E), indicating the immune-suppressive status of CD4^+^ Tregs had been enhanced after dual therapy. These observations suggest that SH100 and OLA dual therapy might be augmented by anti-PD-1 therapy. Therefore, we examined tumor metastasis and immune cells 7 days after primary tumor removal (Additional file 1: Fig. S8A). We found that the PD-1 inhibitor alone was insufficient to reduce the number of lung metastases, whereas triple therapy, which included the PD-1 antibody, could significantly reduce the number of lung metastases, although metastasis was still detectable in the majority of treated mice (Additional file 1: Fig. S8B). To explore the overall immune status inside the treated tumor, we performed immunohistochemical analyses which revealed increased lymphocyte infiltration in the primary tumors after OV therapy (Additional file 1: Fig. S8C). Although the total numbers of CD4^+^ and CD8^+^ T cells did not change after SH100 treatment (Additional file 1: Fig. S8D, E), we found that the percentage of CD8^+^ PD-1^+^ T cells significantly increased after both dual and triple therapy, supporting our rationale for the inclusion of an anti-PD-1 antibody in therapy (Additional file 1: Fig. S8F, G).

To identify the potential mechanism underlying the reduced metastasis rate, we isolated total RNA from primary tumors (*n*=4, control or SH100+OLA+anti-PD-1) 7 days after the last intratumor injection of SH100 and performed a transcriptome analysis. We found that compared with those in the control group, numerous genes in the treated with triple therapy were differentially expressed. Specifically, a total of 81 upregulated genes were identified with an FDR value less than 0.01 and a threefold change (Additional file 1: Figure S9A; Additional file 2: Table S2 and Methods). Surprisingly, we verified the upregulation of immunosuppressive genes via *in vivo* transcriptome analysis (Additional file 1: Figure S9B-G) and *in vitro* analysis (Additional file 1: Figure S9H, I). Interestingly, several upregulated immunosuppressive genes, including *Il-10*, *Il-13*, and *Ccl8*, were involved in the enhanced function of M2 macrophages. The expression of the conventional marker gene of M2 macrophages, *Cd163*, also increased after triple therapy. These results indicated that combined treatment with an anti-PD-1 antibody further improved the antitumor efficacy of SH100 and OLA.

**Optimized triple therapy blocked TNBC metastasis and extended mouse survival**

Although the above results showed that the PD-1 antibody could further enhance the efficacy of OV and OLA dual therapy, we still found lung metastasis in nearly all the mice, which might be due to delayed PD-1 antibody administration (5 days after oncolytic therapy). Therefore, we modified the regimen by injecting PD-1 antibodies one day after SH100 administration (Additional file 1: Fig. S10A). We found that the PD-1 antibody alone had no effect on the size of the tumor *in situ*, whereas both the dual therapy of SH100 and OLA and the modified triple therapy of SH100, OLA and PD-1 antibody significantly suppressed the growth of primary tumors (Additional file 1: Fig. S10B) without causing significant weight changes in mice at the time of primary tumor resection (Additional file 1: Fig. S10C). Notably, according to the optimized protocol, compared with no treatment, triple therapy significantly increased the survival rate (*P*=0.0369), and 60% of the mice survived 100 days after treatment without metastasis (Additional file 1: Fig. S10D). In contrast, nearly all the mice in the PD-1 monotherapy and dual therapy groups died of lung metastasis (Additional file 1: Fig. S10D).

Next, we evaluated the efficacy of triple therapy in another TNBC lung metastasis model using AT3 cells [15] (Fig. 2F). We found that both dual therapy and triple therapy significantly reduced the size of the primary tumors compared with those in the nontreated control group and the anti-PD-1 monotherapy group (Fig. 2G). Like in the 4T1 model, we found that both dual and triple therapy could alleviate lung metastasis in the AT3 model, but there were no significant differences in lung bioluminescence imaging (BLI) between the two groups. This difference is likely due to AT3 being less metastatic than 4T1 (Fig. 2H). Although SH100 treatment led to slight weight loss in the AT3 model, the weights of the mice returned to normal two weeks after treatment (Fig. 2I). Notably, the survival rate of mice treated with triple therapy increased from 33.33% after dual therapy to 55.56%, indicating the benefit of PD-1 antibody inclusion (Fig. 2J).

To further verify the effectiveness of combination therapy, we conducted the same experiment in a melanoma model, named B16F10n-1 (Additional file 1: Fig. S10E). Although we found a significant reduction in tumor volume in the oncolytic groups treated with SH100 monotherapy or in combination with SH100 (Additional file 1: Fig. S10F), there was a notable difference in the number of mice with complete visible tumor clearance; 2 mice were treated with SH100 alone, 2 mice were treated with dual therapy, and 5 mice were treated with triple therapy. Moreover, we found no significant difference in body weight among the groups, which indicated the acceptable safety of the treatment regimens (Additional file 1: Fig. S10G). Consistent with the number of mice with complete tumor clearance, 55.56% of the mice survived in the triple therapy group, whereas only 25.00% of the mice survived in the SH100 monotherapy and dual therapy groups (Additional file 1: Fig. S10H).

**Combination therapy established long-term tumor-specific immunological memory and systemic antitumor immunity**

To examine tumor-specific immunological memory, the previously cured mice in Fig. 2J were rechallenged with the same type of tumor cells (Fig. 2K). As shown in Fig. 2L, all the mice in the naïve group were tumor-positive 14 days after AT3 inoculation, whereas most of the mice in the dual therapy and triple therapy groups were tumor-free except for one in the triple therapy group, likely due to T-cell exhaustion. We then assessed tumor-specific T cells by interferon-γ (IFN-γ) enzyme-linked immunospot (ELISPOT) analysis. Our data showed that both combination therapies induced a strong T-cell response against AT3 cells in tumor-free mice, indicating that oncolytic treatment could establish long-term tumor-specific immunological memory and systemic antitumor immunity (Fig. 2M). Interestingly, after treating tumor bearing mice from the triple therapy with PD-1 antibody alone four times, we found that the tumor disappeared within 2 weeks, suggesting that the antitumor function of the exhausted tumor-specific T cells could be restored.

To understand systemic antitumor immunity, we collected splenocytes from the naïve group and triple therapy group (*n* = 3 mice per group) and used cytometry by time of flight (CyTOF) to analyze the immune microenvironment (Additional file 1: Fig. S11A). Our results revealed that the total T cells, CD4^+^ T cells, and CD8^+^ T cells cell counts were significantly greater in the triple therapy group than in the naïve group, which indicated an active immune status (Additional file 1: Fig. S11B). In contrast, the percentage of B cells slightly decreased (Additional file 1: Fig. S11B). CD8^+^ T cells usually play a key role in the long-term tumor-specific T cell response, so we analyzed subclusters of CD8^+^ T cells (Additional file 1: Fig. S11C). Consistent with the ELISPOT results shown in Fig. 2L, the proportion of C05_Ly6c^+^ naïve CD8 T cells decreased in splenocytes from the triple therapy group that had rejected AT3 tumors after rechallenge, while the proportions of C08_CD86^-^, C09_CD11c^+^ central memory CD8 T cells, and C12_CD27^+^CD86^+^Ly6c^-^ effector memory CD8 T cells increased (Additional file 1: Fig. S11D). Next, we detected the expression of exhaustion markers (CTLA-4, PD-1, LAG-3, and TIM-3) in the two groups and found that no significant difference existed except for a slight decrease in CTLA-4 after triple therapy (Additional file 1: Fig. S11E). Then, we analyzed the changes in subclusters of CD4^+^ T cells among the splenocytes (Additional file 1: Fig. S12A), and found that only C09_Ly6c^-^FR4^+^CD86^+^ naïve CD4 T cells were significantly reduced (Additional file 1: Fig. S12B). Furthermore, the proportion of CD4^+^ Tregs was lower in the triple therapy group than in the naïve group (Additional file 1: Fig. S12C). These results indicate that the treatment of tumor-bearing mice with triple combination therapy established long-term tumor-specific immunological memory and systemic antitumor immunity.

**Supplemental Discussion**

Compared to other immunotherapy methods, the major advantage of OVs lies in their ability to effectively infect and rapidly lyse tumor cells, thereby releasing a substantial amount of tumor-associated antigens (TAAs) and transforming the initially "cold" tumor microenvironment into an inflamed or "hot" state [16]. Additionally, OVs can activate innate immune responses through diverse signaling pathways, thus augmenting the overall antitumor immune status. The safety and oncolytic efficacy of OVs are two crucial determinants of their clinical application [17]. Previous studies have predominantly employed direct deletion of the HSV-1 *ICP34.5* and *ICP47* genes [6, 7]; however, this approach has been associated with a reduction in viral proliferation capacity and an impact on oncolytic potential. In this study, we precisely regulated the expression of the virulence gene *ICP34.5* by utilizing *miR124T* while simultaneously introducing the inflammatory cytokine *GM-CSF* at this specific genomic locus. The one-step growth curves demonstrated that the modified SH100 strain exhibited only a slight decrease in productivity compared to its parental strain HSV-1 KOS. Furthermore, safety tests revealed a limited number of SH100 in the TG and brain, which are two primary sites associated with latent HSV-1 infection. These results demonstrated that our modified OV not only enhances the safety profile but also augments the proliferative capacity.

A major challenge in the current practical application of OVs is their recognition and elimination by the immune system upon injection into the body, which leads to a limited presence within tumors and a reduced yield of viral progeny particles, thereby restricting the therapeutic efficacy of OVs [18-20]. Consequently, it is imperative to systematically explore inhibitors that can promote viral replication. Through CRISPR screening, we identified PARP1 as a potential restrictive factor for HSV-1 infection. PARPs are intracellular poly(ADP-ribose) polymerases that catalyze ADP-ribosylation, a posttranslational covalent process involving the transfer of single or multiple subunits of ADP-ribose from NAD^+^ to an acceptor protein [10, 11]. Several researchers have reported that PARP1 inhibits the replication of Epstein Barr Virus (EBV) and Hepatitis B virus (HBV) [21, 22], whereas other studies have demonstrated its potential to enhance the replication of Japanese encephalitis virus (JEV) and Pseudorabies Virus (PRV) [23, 24]. In this study, we observed that the inhibition of PARP1 using either shRNA or OLA significantly enhanced SH100 replication in tumor cells, while the knockdown of PARP2 did not have the same effect. These findings suggest that PARP1 is the primary antiviral PARP involved in oncolytic HSV-1 infection. Additionally, to further substantiate this conclusion, we conducted virus replication experiments on diverse tumor cells and immune cells and found that OLA effectively enhanced HSV-1 replication across various cell types. Moreover, we also confirmed this effect in three different tumor models, demonstrating that OLA could facilitate the replication of SH100 within tumors. A recent study showed that PARP1 modifies cyclic GMP-AMP synthase (cGAS) in the innate immune signaling pathway by PARylation, thus preventing cGAS from recognizing viral DNA and inhibiting the innate immune responses, whereas the addition of OLA can inhibit the binding of PARP1 to cGAS, thereby enhancing the innate immune response and inhibiting viral replication [14]. Wild-type THP-1 cells have a complete innate immune signaling pathway and are sensitive to viruses. PARP1 can negatively regulate the innate immune response through cGAS to promote virus replication. The addition of OLA weakens PARP1's ability to negatively regulate the innate immune response, leading to a reduction in viral replication. Instead, in THP-1 KO-STING cells, due to the absence of the innate immune signaling pathway, PARP1 cannot promote virus replication through inhibiting the innate immune response, in which case PARP1 plays a restrictive role in virus replication through a separate mechanism independent of the innate immune response. Therefore, when OLA is added to cGAS-STING nonfunctional cells, the restrictive role of PARP1 is inhibited, and virus replication is increased. Consistent with previous findings, our results indicate that cotreatment with OLA can effectively reduce viral replication in wild-type THP-1 cells but not in STING-knockout THP-1 cells, suggesting that the suppressive effect of PARP1 is dependent on the compromised state of the innate immune response, which is common in tumor cells. Additionally, we noted that studies have explored the potential synergistic effect of combining PARP inhibitors with various types of oncolytic viruses for enhanced tumor eradication. Ning et al. reported that the PARP inhibitor OLA and oHSV synergized to improve the efficacy of eradicating glioma stem cells; however, the study did not reveal that PARP1 has a restrictive effect on HSV-1 replication. [25]. Passaro et al. reported that OLA could enhance the oncolytic effect of AdV on anaplastic thyroid carcinoma by promoting AdV replication [26]. Both AdV and HSV-1 are double-stranded DNA viruses, and PARP1 can bind DNA. By inhibiting PARP1 activity, OLA may attenuate the binding affinity of PARP1 to the DNA virus genome, thereby promoting viral replication.

Based on the *in vitro* viral replication results, we devised a combination strategy involving SH100 and OLA. Our study demonstrated that the combination of SH100 and OLA significantly prolonged the overall survival of mice in a mouse GBM model, whereas neither SH100 nor OLA alone was able to achieve this effect. However, it was noteworthy that the survival curve of mice in the SH100-only group did not significantly differ from that of mice in the control group, potentially attributed to the limited viral dosage administered, underlying the rationality of combining SH100 with OLA for treatment. TNBC, characterized by strong metastatic activity, particularly lung metastasis, is considered the most lethal subtype of breast cancer and has the lowest overall survival rate among all types [27, 28]. Although PARP inhibitors have gained approval for TNBC treatment, their overall efficacy remains inconclusive, possibly due to the potential induction of protumor macrophages [29]. In the present study, SH100 alone significantly reduced the incidence of lung metastasis in the mouse TNBC model 4T1. Furthermore, when SH100 was combined with OLA, its efficacy was enhanced by further reducing the number of lung metastases. Conversely, OLA monotherapy did not have any substantial therapeutic effect. Through scRNA-seq, we identified the upregulation of multiple immune checkpoint molecules on CD4^+^ Treg cells in lung metastases, indicating increased sensitivity to ICIs following combined treatment with SH100 and OLA. Consequently, we introduced an anti-PD-1 Ab as a therapeutic intervention. Our findings demonstrated that the addition of an anti-PD-1 antibody further augmented the therapeutic efficacy of SH100 and OLA, leading to a greater reduction in lung metastases. Moreover, we conducted a preliminary analysis of the immune microenvironment within the primary tumor and observed that virus injection induced significant aggregation of CD45^+^ cells in the tumor, transforming the tumor from a cold to a hot state. However, it is noteworthy that certain immunosuppressive genes exhibited varying degrees of upregulation within the tumor, indicating potential limitations on the efficacy of PD-1 Ab when administered too late. Therefore, we advanced the use of PD-1 Ab by increasing the dosage and extending the treatment duration. The efficacy of the optimized regimen was validated in two mouse models of TNBC. Triple therapy significantly inhibited lung metastasis and prolonged survival in the mice. Our findings demonstrated that both dual therapy and triplet therapy produced robust and specific antitumor T-cell immune responses, as evidenced by the enhanced secretion of IFN-γ, elevated proportions of CD8^+^ Tcm and CD8^+^ Tem cells, and a reduced proportion of naïve CD8^+^ T cells. Our study thus demonstrated that the combination of OLA and PD-1 Ab synergistically facilitates the establishment of durable tumor-specific immune memory via OV, leading to a robust systemic antitumor immune response and ultimately enhancing overall therapeutic efficacy.

Our study has several limitations. Although we have demonstrated the ability of OLA to enhance HSV-1 replication *in vitro* and *in vivo*, and the improved efficacy of SH100 in combination with OLA in GBM and TNBC models, further investigations are needed to elucidate the molecular mechanism underlying the inhibition of tumor metastasis. Furthermore, while we optimized HSV-1 for safety and an augmented immune response, no modifications were made to enhance its oncolytic ability, which could limit the efficacy of SH100. Therefore, future efforts may focus on optimizing SH100 to augment the oncolytic ability of the virus such as by introducing fusogenic glycoproteins to facilitate the spread of OV in the tumor microenvironment. Additionally, exploring the potential application of SH100 and OLA in the treatment of diverse tumor types while judiciously employing PD-1 Abs based on individual tumor characteristics is imperative. Furthermore, although the survival rate of the triple therapy group was higher than that of the dual therapy group, the difference was statistically insignificant between the two groups, which may be related to the relatively small number of mice in each group.

In summary, we developed an OV based on HSV-1 and demonstrated its efficacy as an immunotherapeutic strategy for cancer treatment. Systematic discovery facilitated us to design a triple combination therapy with an OV that effectively overcame multiple constraints and significantly enhanced the efficacy of tumor treatment. Our study provides a solid foundation for future studies to explore strategies to block these suppressive effects, which may further enhance the clinical efficacy of oncolytic therapy.

**References**

1. Desai P, Person S. Incorporation of the green fluorescent protein into the herpes simplex virus type 1 capsid. J Virol. 1998;72(9):7563-8.

2. Rosato PC, Katzenell S, Pesola JM, North B, Coen DM, Leib DA. Neuronal ifn signaling is dispensable for the establishment of hsv-1 latency. Virology. 2016;10(497):323-7.

3. Miller CG, Krummenacher C, Eisenberg RJ, Cohen GH, Fraser NW. Development of a syngenic murine b16 cell line-derived melanoma susceptible to destruction by neuroattenuated hsv-1. Mol Ther. 2001;3(2):160-8.

4. Jackson JW, Hall BL, Marzulli M, Shah VK, Bailey L, Chiocca EA, et al. Treatment of glioblastoma with current ohsv variants reveals differences in efficacy and immune cell recruitment. Mol Ther Oncolytics. 2021;29(22):444-53.

5. Orvedahl A, Alexander D, Talloczy Z, Sun Q, Wei Y, Zhang W, et al. Hsv-1 icp34.5 confers neurovirulence by targeting the beclin 1 autophagy protein. Cell Host Microbe. 2007;1(1):23-35.

6. Harrington KJ, Puzanov I, Hecht JR, Hodi FS, Szabo Z, Murugappan S, et al. Clinical development of talimogene laherparepvec (t-vec): a modified herpes simplex virus type-1-derived oncolytic immunotherapy. Expert Rev Anticancer Ther. 2015;15(12):1389-403.

7. Thomas S, Kuncheria L, Roulstone V, Kyula JN, Mansfield D, Bommareddy PK, et al. Development of a new fusion-enhanced oncolytic immunotherapy platform based on herpes simplex virus type 1. J Immunother Cancer. 2019;7(1):214.

8. Jia X, Wang X, Guo X, Ji J, Lou G, Zhao J, et al. Microrna-124: an emerging therapeutic target in cancer. Cancer Med. 2019;8(12):5638-50.

9. Sun Y, Luo ZM, Guo XM, Su DF, Liu X. An updated role of microrna-124 in central nervous system disorders: a review. Front Cell Neurosci. 2015;9:193.

10. Zada D, Sela Y, Matosevich N, Monsonego A, Lerer-Goldshtein T, Nir Y, et al. Parp1 promotes sleep, which enhances dna repair in neurons. Mol Cell. 2021;81(24):4979-93.

11. Alemasova EE, Lavrik OI. Poly(adp-ribosyl)ation by parp1: reaction mechanism and regulatory proteins. Nucleic Acids Res. 2019;47(8):3811-27.

12. Curtin NJ, Szabo C. Poly(adp-ribose) polymerase inhibition: past, present and future. Nat Rev Drug Discov. 2020;19(10):711-36.

13. Minn AJ, Gupta GP, Siegel PM, Bos PD, Shu W, Giri DD, et al. Genes that mediate breast cancer metastasis to lung. Nature. 2005;436(7050):518-24.

14. Wang F, Zhao M, Chang B, Zhou Y, Wu X, Ma M, et al. Cytoplasmic parp1 links the genome instability to the inhibition of antiviral immunity through parylating cgas. Mol Cell. 2022;82(11):2032-49.

15. Xiao Y, Cong M, Li J, He D, Wu Q, Tian P, et al. Cathepsin c promotes breast cancer lung metastasis by modulating neutrophil infiltration and neutrophil extracellular trap formation. Cancer Cell. 2021;39(3):423-37.

16. Martin NT, Bell JC. Oncolytic virus combination therapy: killing one bird with two stones. Mol Ther. 2018;26(6):1414-22.

17. Jayawardena N, Poirier JT, Burga LN, Bostina M. Virus-receptor interactions and virus neutralization: insights for oncolytic virus development. Oncolytic Virother. 2020;6(9)1-15.

18. Nemunaitis J, Senzer N, Sarmiento S, Zhang YA, Arzaga R, Sands B, et al. A phase i trial of intravenous infusion of onyx-015 and enbrel in solid tumor patients. Cancer Gene Ther. 2007;14(11):885-93.

19. Kirn D. Clinical research results with dl1520 (onyx-015), a replication-selective adenovirus for the treatment of cancer: what have we learned? Gene Ther. 2001;8(2):89-98.

20. Quillien L, Top S, Kappler-Gratias S, Redoute A, Dusetti N, Quentin-Froignant C, et al. A novel imaging approach for single-cell real-time analysis of oncolytic virus replication and efficacy in cancer cells. Hum Gene Ther. 2021;32(3-4):166-77.

21. Lupey-Green LN, Moquin SA, Martin KA, Mcdevitt SM, Hulse M, Caruso LB, et al. Parp1 restricts epstein barr virus lytic reactivation by binding the bzlf1 promoter. Virology. 2017;507:220-30.

22. Ko HL, Ng HJ, Goh EH, Ren EC. Reduced adp-ribosylation by parp1 natural polymorphism v762a and by parp1 inhibitors enhance hepatitis b virus replication. J Viral Hepat. 2013;20(9):658-65.

23. Desingu PA, Mishra S, Dindi L, Srinivasan S, Rajmani RS, Ravi V, et al. Parp1 inhibition protects mice against japanese encephalitis virus infection. Cell Rep. 2023;42(9):113103.

24. Li GL, Ding GX, Zeng L, Ming SL, Fu PF, Wang Q, et al. Inhibition of parp1 dampens pseudorabies virus infection through dna damage-induced antiviral innate immunity. J Virol. 2021;95(16):e76021.

25. Ning J, Wakimoto H, Peters C, Martuza RL, Rabkin SD. Rad51 degradation: role in oncolytic virus-poly (adp-ribose) polymerase inhibitor combination therapy in glioblastoma. J Natl Cancer Inst. 2017;109(3):1-13.

26. Passaro C, Volpe M, Botta G, Scamardella E, Perruolo G, Gillespie D, et al. Parp inhibitor olaparib increases the oncolytic activity of dl922-947 in in vitro and in vivo model of anaplastic thyroid carcinoma. Mol Oncol. 2015;9(1):78-92.

27. Trivers KF, Lund MJ, Porter PL, Liff JM, Flagg EW, Coates RJ, et al. The epidemiology of triple-negative breast cancer, including race. Cancer Causes Control. 2009;20(7):1071-82.

28. Cinkaya A, Akin M, Sengul A. Evaluation of treatment outcomes of triple-negative breast cancer. J Cancer Res Ther. 2016;12(1):150-4.

29. Mehta AK, Cheney EM, Hartl CA, Pantelidou C, Oliwa M, Castrillon JA, et al. Targeting immunosuppressive macrophages overcomes parp inhibitor resistance in brca1-associated triple-negative breast cancer. Nat Cancer. 2021;2(1):66-82.

**Supplemental Figures**


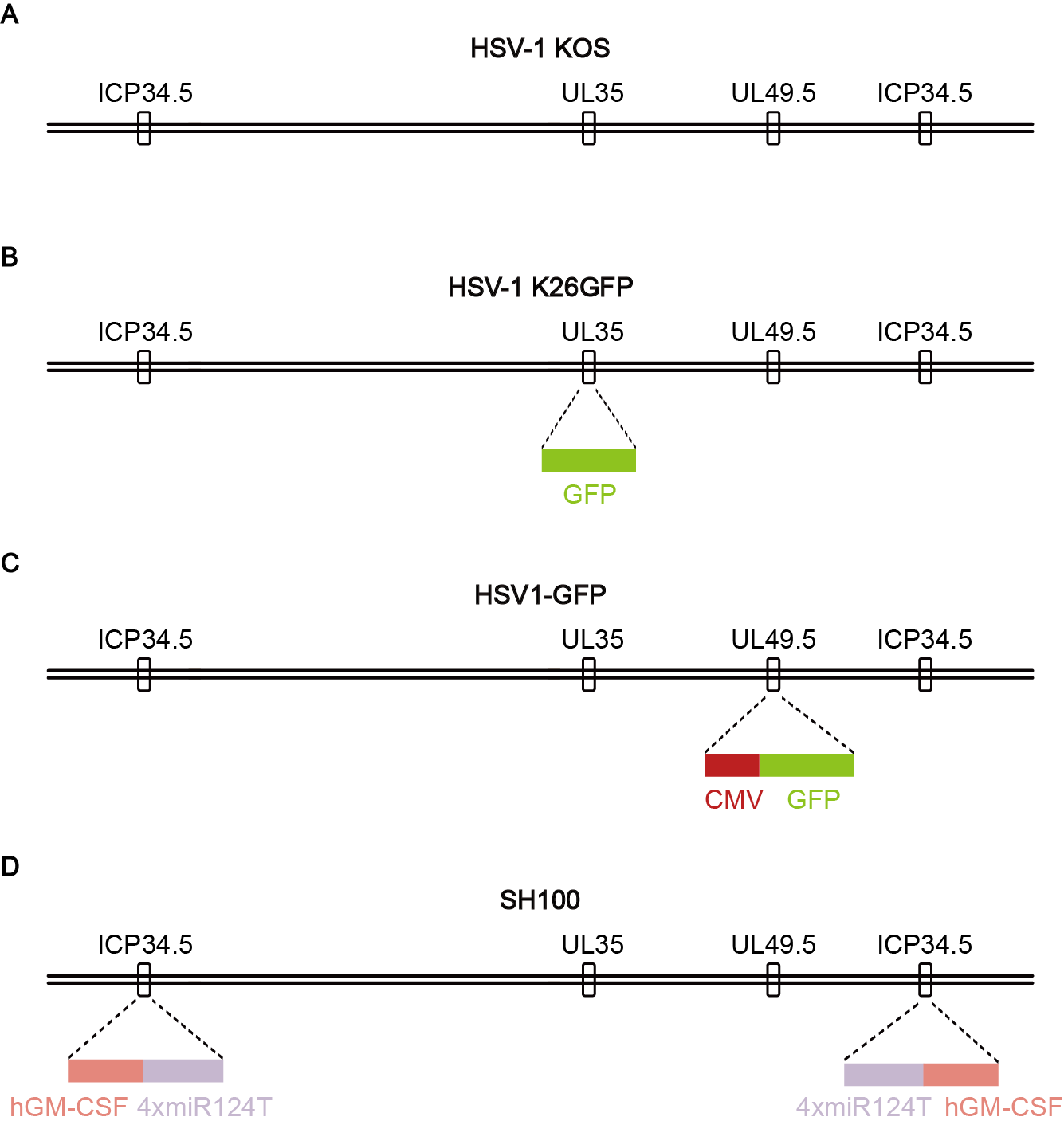


**Fig. S1. Diagram of the virus structure.** The structural diagrams of HSV-1 KOS (**A**), HSV-1-K26GFP (**B**), HSV1-GFP (**C**), and SH100 (**D**).


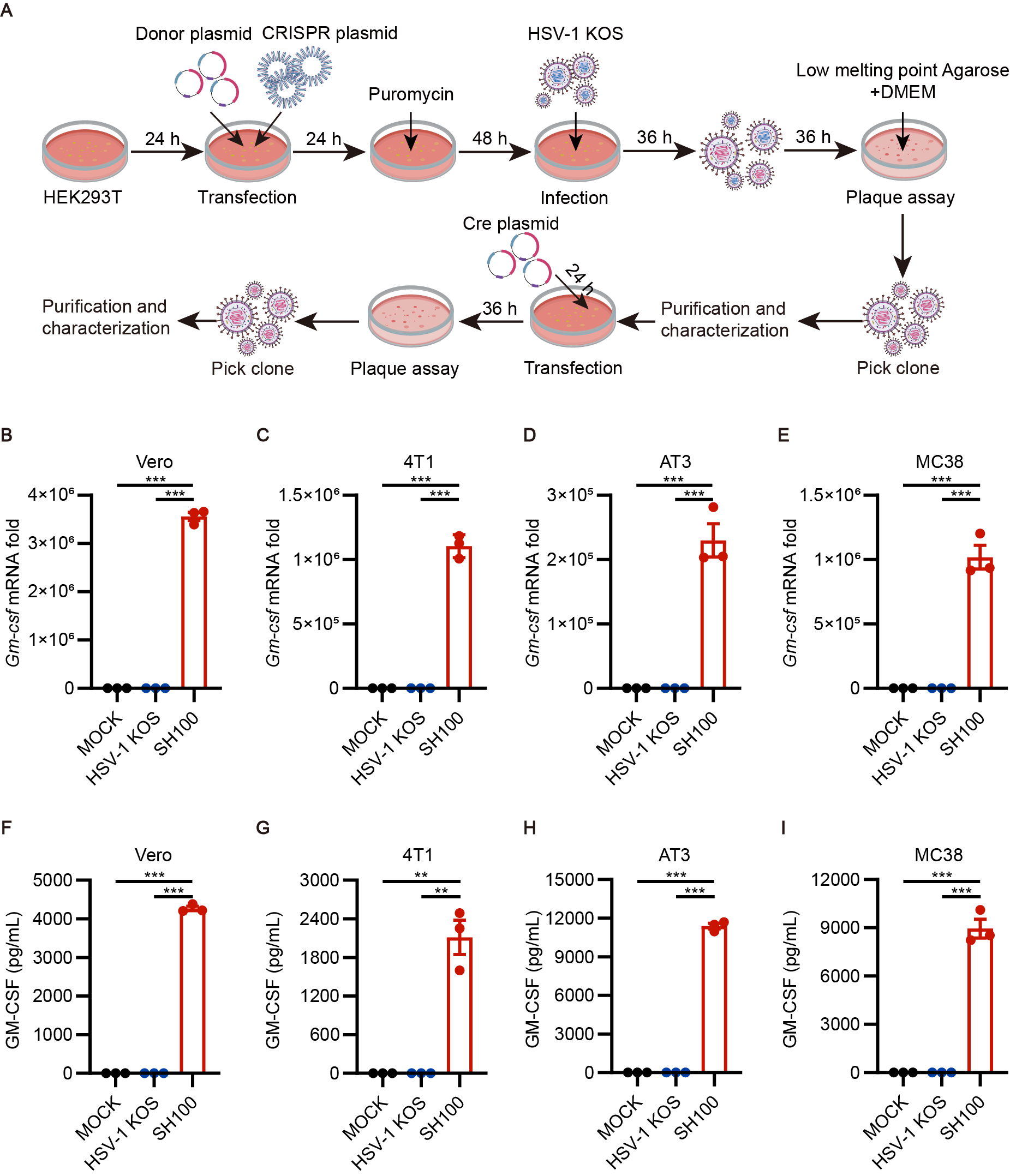


**Fig. S2. Generation and characterization of a neuron-detargeted recombinant oncolytic HSV-1.** **A** Schematic diagram illustrating steps for construction of oncolytic virus strain SH100. **B-I** Evaluation of the expression of *Gm-csf* inserted into the SH100 genome in different cell lines. Cells were infected with HSV-1 KOS or SH100 (MOI=0.8) for 24 h (*n*=3), followed by RT-qPCR (**B-E**) and ELISA (**F-I**). *P* values were obtained by unpaired two-tailed *t* test (**B-I**), ^**^*P*<0.01; ^***^*P*<0.001. Data were shown as the means ± SEM.


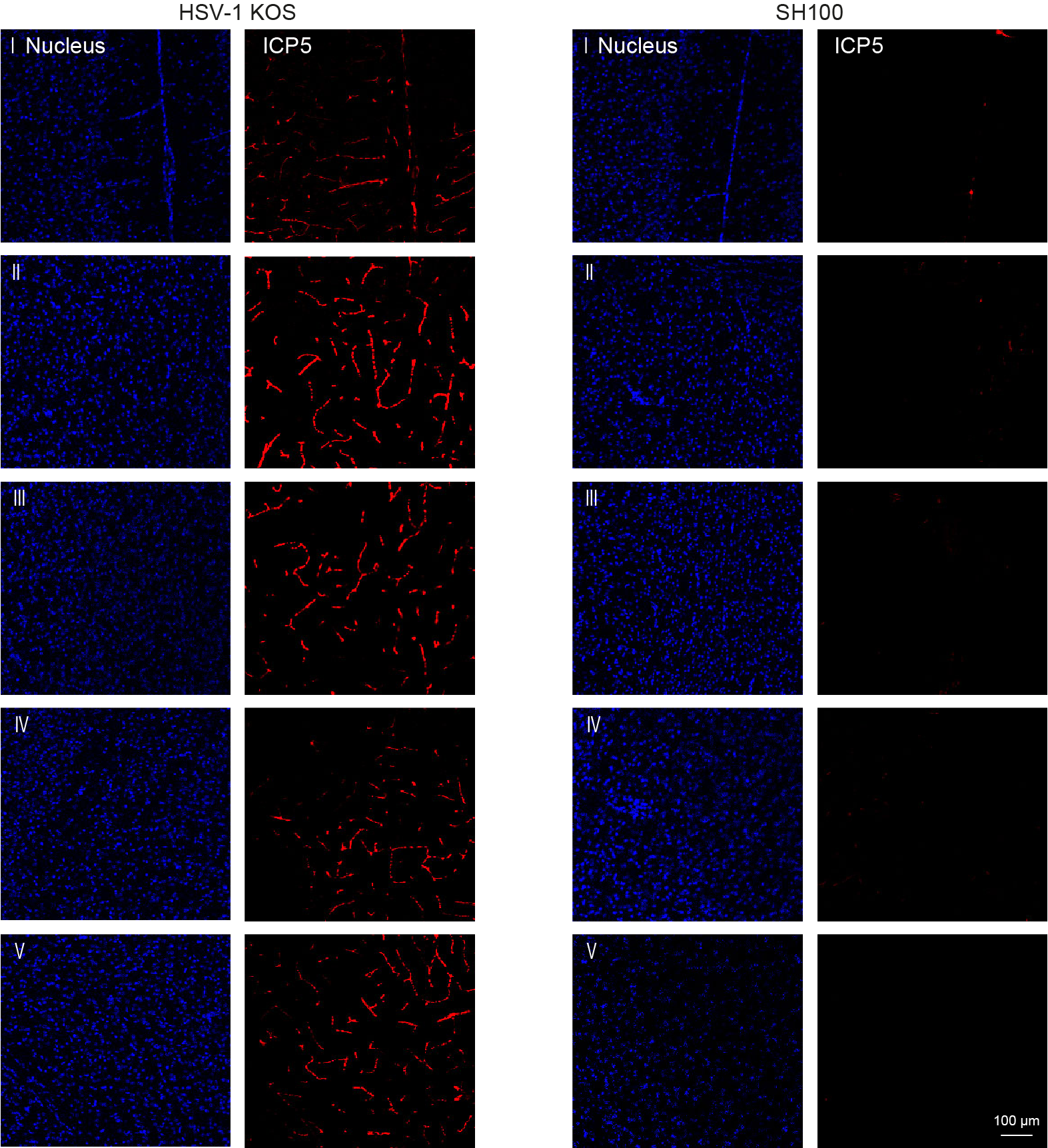


**Fig. S3. Safety analysis of SH100 in mice.** Nine days after infection, mice were euthanized, and the distribution of viruses in the brain was detected by immunofluorescence. The magnified images of different regions were linked to Fig. 1D, I, Cortex; II &III, septal nucleus; IV&V, striatum. Mice were infected with SH100 or HSV-1 KOS at a dose of 2×10^6^ PFU/eye.


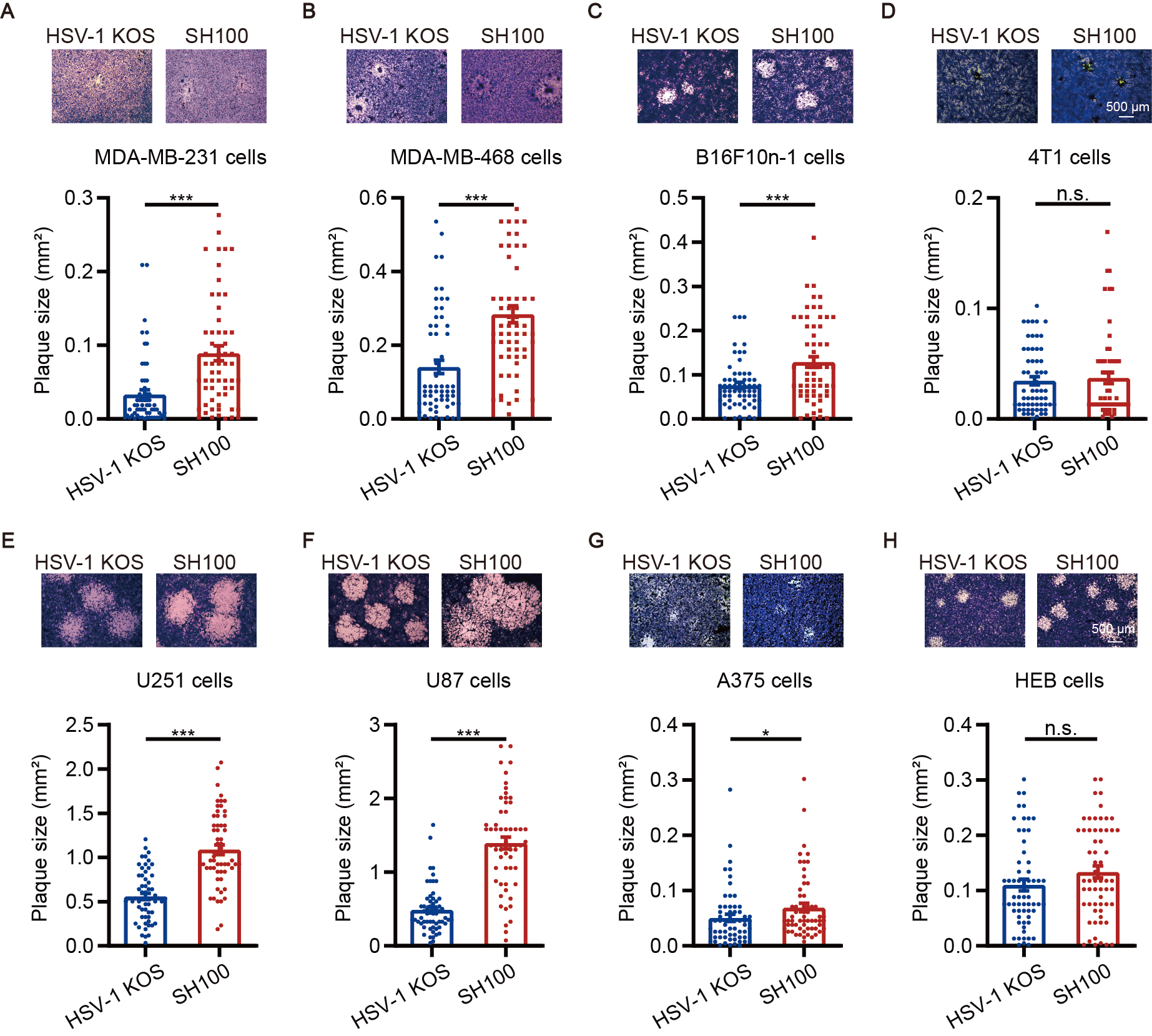


**Fig. S4. Evaluation of the oncolytic activity of SH100 *in vitro***. Evaluation of the oncolytic activity of SH100 in different tumor cell lines by crystal violet staining (MOI=0.01). *P* values were obtained by unpaired two-tailed *t* test (**A-H**), n.s., non-significant; ^*^*P*<0.05; ^***^*P*<0.001. Data were shown as the means ± SEM.


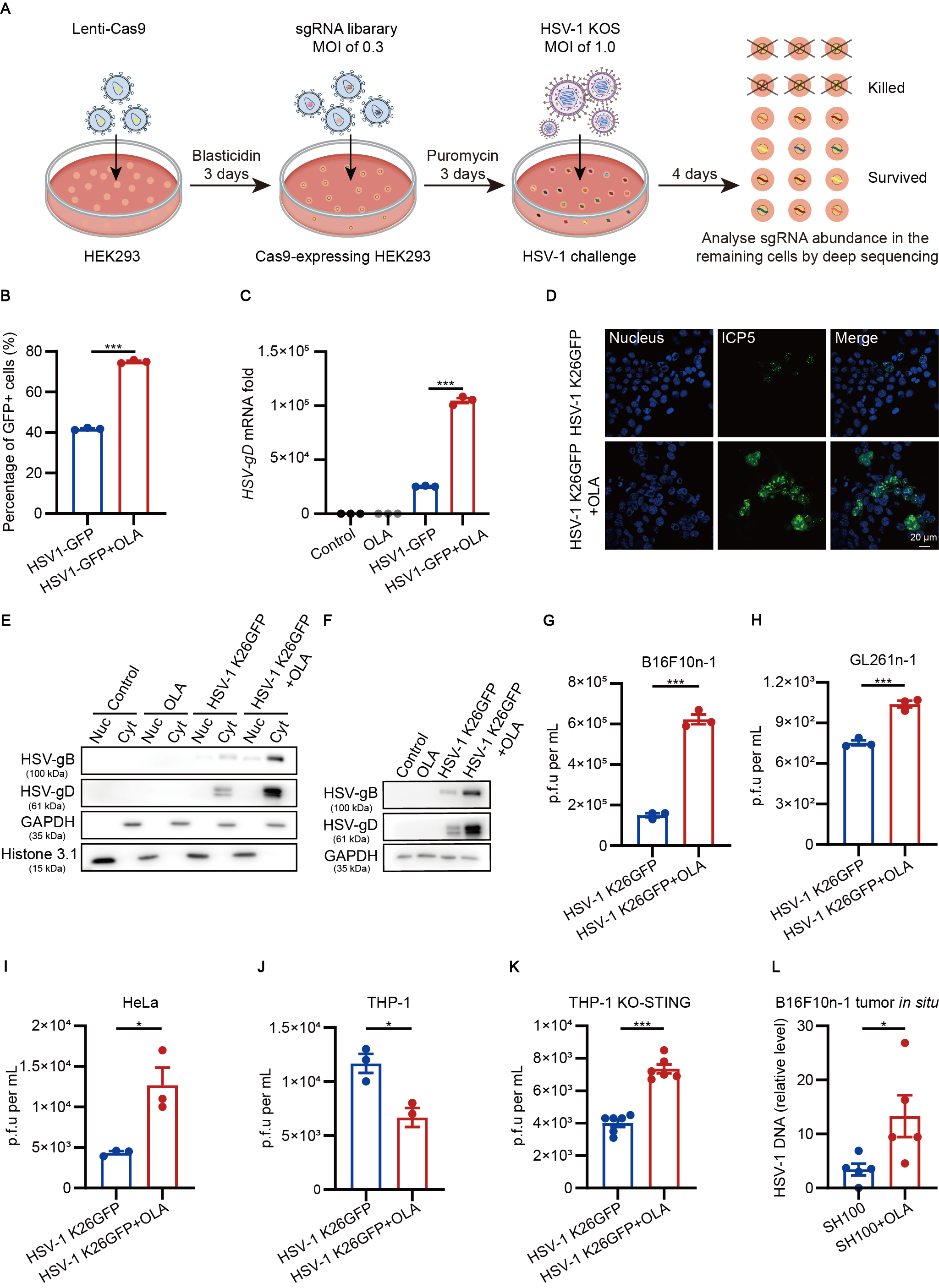


**Fig. S5. Genome-wide CRISPR screening identified intrinsic OV restriction factor PARP1.** **A** Schematic illustration of the CRISPR screening. **B**, **C** 4T1 cells were incubated with 100 µM Olaparib (OLA) for 12 h before infection with HSV1-GFP (MOI=0.2) for an additional 24 h (*n*=3), followed by flow cytometry analysis (**B**) and RT-qPCR (**C**). **D-F** 4T1 cells were treated with 100 µM OLA for 12 h and infected with HSV-1 K26GFP (MOI=0.8) for an additional 24 h (*n*=3), followed by confocal microscopy (**D**) and Western blot (**E**, **F**). Western blot analysis of the HSV-1 gB and gD in the nuclear and cytoplasmic fractions (**E**), and whole cell sample (**F**). Histone 3.1 and GAPDH were used as an internal control of nucleus and cytoplasm, respectively. Nuc and Cyt indicated nucleus and cytoplasm, respectively. **G-I** Tumor cells were treated with 100 µM OLA for 12 h and infected with HSV-1 K26GFP (MOI=0.8) for additional 24 h (*n*=3), followed by plaque assay. **J**, **K** THP-1 and THP-1 KO-STING cells were pretreated with 100 ng/mL PMA for 48 h before adding 100 µM OLA for 12 h. Cells were then infected with HSV-1 K26GFP (MOI=0.8) for an additional 24 h (*n*=3 or 6), followed by plaque assay. **L** Mice received OLA or PBS *i.p.* for 3 days, followed by intratumor injection of SH100 (5 × 10^7^ PFU per mouse). After 2 days, tumors were harvested and virus load was detected by RT-PCR of HSV-1 genomic DNA. *n*=5 per each group. *P* values were obtained by unpaired two-tailed *t* test (**B, C, G-L**), ^*^*P*<0.05; ^***^*P*<0.001. Data were shown as the means ± SEM.

**
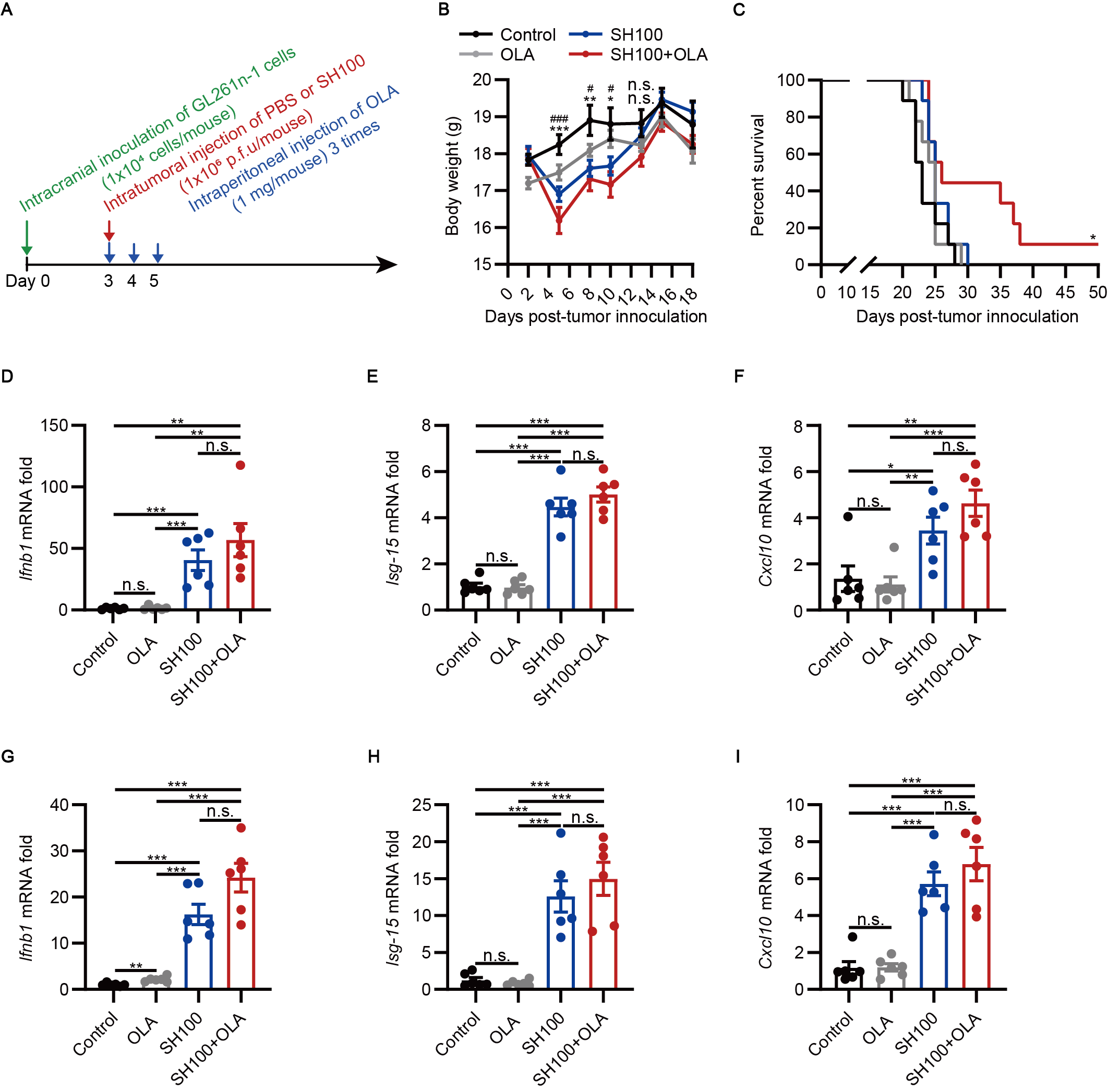
**

**Fig. S6. Analysis of innate immune responses in the primary tumors.** **A** Schematic illustration of the work plan for SH100 and OLA dual therapy in the treatment of GL261n-1 tumors. **B, C** Evaluation the efficacy of dual therapy in GL261n-1 tumors. Body weight (**B**); Kaplan-Meier survival curves of C57BL/6J mice (**C**) (*n*=9 mice per group). **D-I** Evaluation of the innate immune response within the 4T1 primary tumors (**D-F**) and the AT3 primary tumors (**G-I**). Mice received OLA or PBS intraperitoneal injection (i.p.) for 3 days, followed by intratumor injection (i.t.) of SH100 (5 × 10^7^ PFU per mouse). After 24 h, tumors were collected, followed by RT-qPCR (*n*= 6 mice per group). *P* values were obtained by unpaired two-tailed *t* test (**B**, **D-I**), n.s., non-significant; ^*, #^*P*<0.05; ^**^*P*<0.01; ^***, ###^*P*<0.001, or Mantel-Cox test (**C**), ^*^*P*<0.05. Data were shown as the means ± SEM. In (**B**), the symbol “^*^” denotes the difference between Control and SH100+OLA; the symbol “^#^” denotes the difference between Control and SH100.


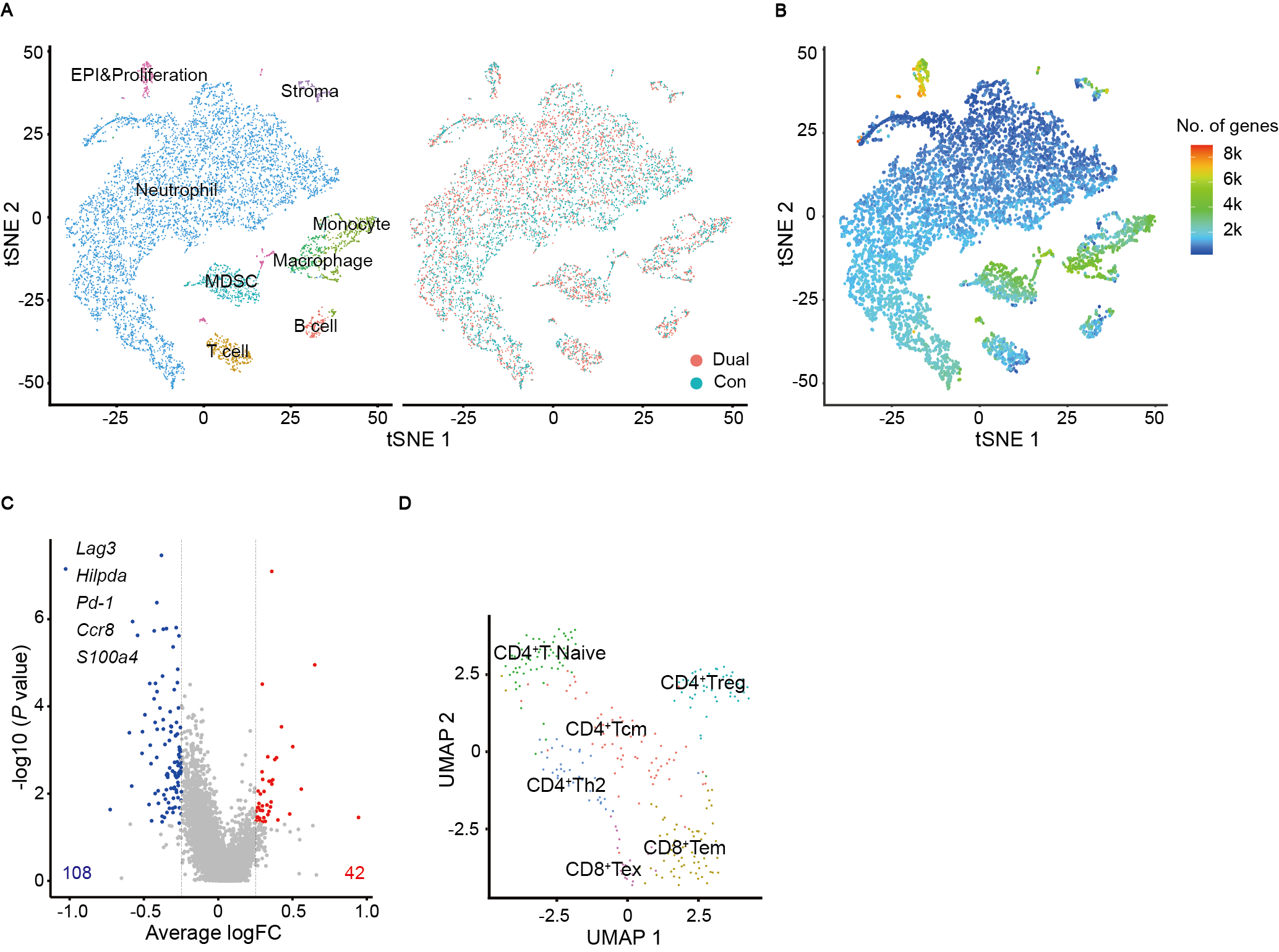


**Fig. S7. Single-cell RNA sequencing was utilized to elucidate the tumor microenvironment within lung metastases. A** A t-distributed stochastic neighbor embedding (tSNE) view of 8,437 single cells, color-coded by the assigned cell types. **B** A tSNE view of all cells, color-coded by the number of genes detected in each cell. **C** Volcano plot for the distribution trends for DEGs in all T cells between the control group and the dual therapy group. The blue color dots represent downregulated genes. The red color dots represent upregulated genes. **D** UMAP view of T cells, colored-coded by re-evaluated cluster.


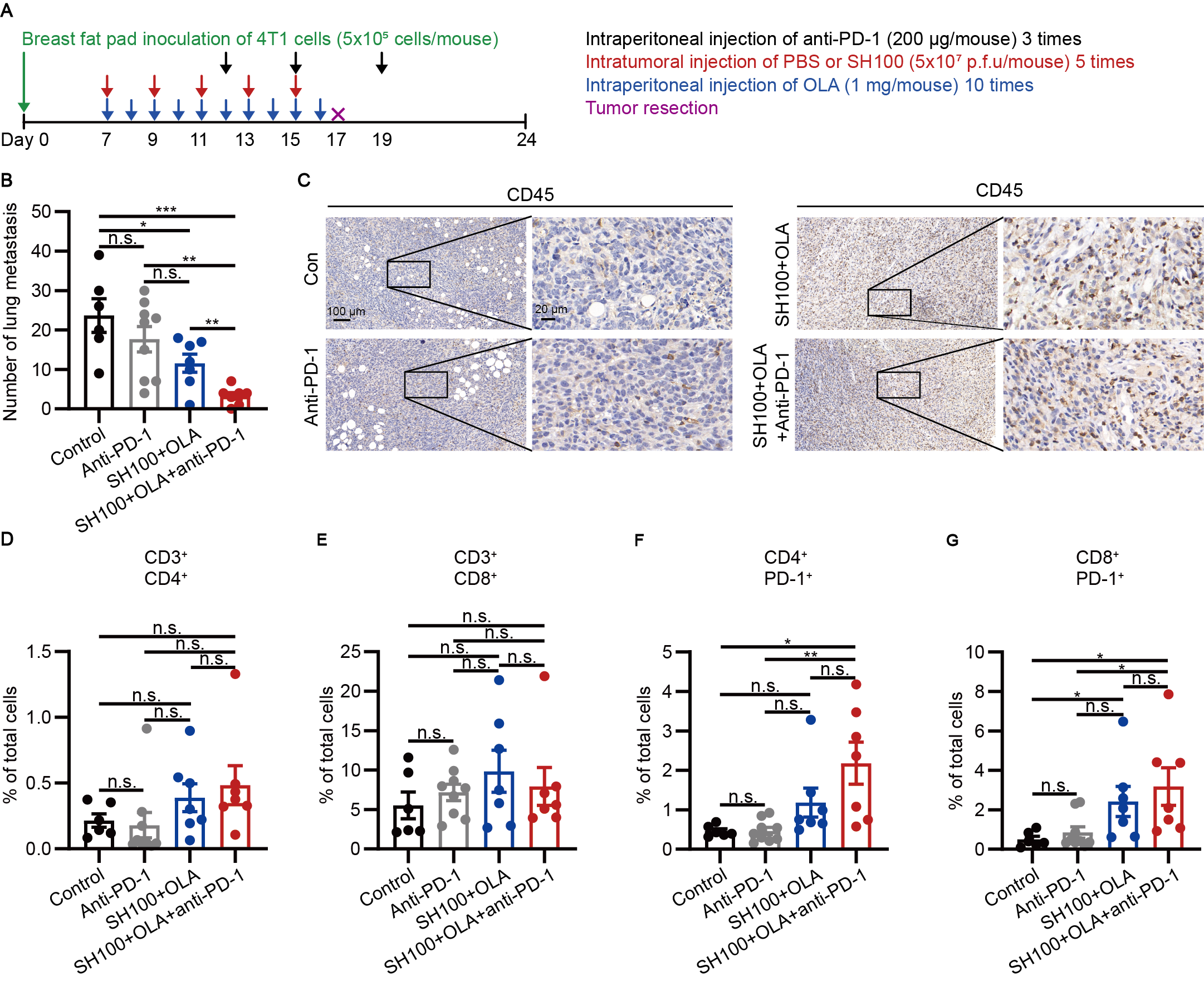


**Fig. S8. Dual therapy with OV and Olaparib sensitizes 4T1 tumors to immune checkpoint inhibitor. A** Schematic illustration of the work plan. **B** The number of lung metastasis. **C** Immunohistochemical analysis of CD45^+^ cells in 4T1 tumors. **D-G** Flow cytometry analysis of respective T cell populations as a percent of total cells in the 4T1 tumors, 1 day after the last injection of OLA. Control, *n*=6; Anti-PD-1, *n*=9; SH100 + OLA, SH100 + OLA + anti-PD-1, *n*=7. *P* values were obtained by unpaired two-tailed *t* test (B and D-G), n.s., non-significant; ^*^*P*<0.05; ^**^*P*<0.01. Data were shown as the means ± SEM.


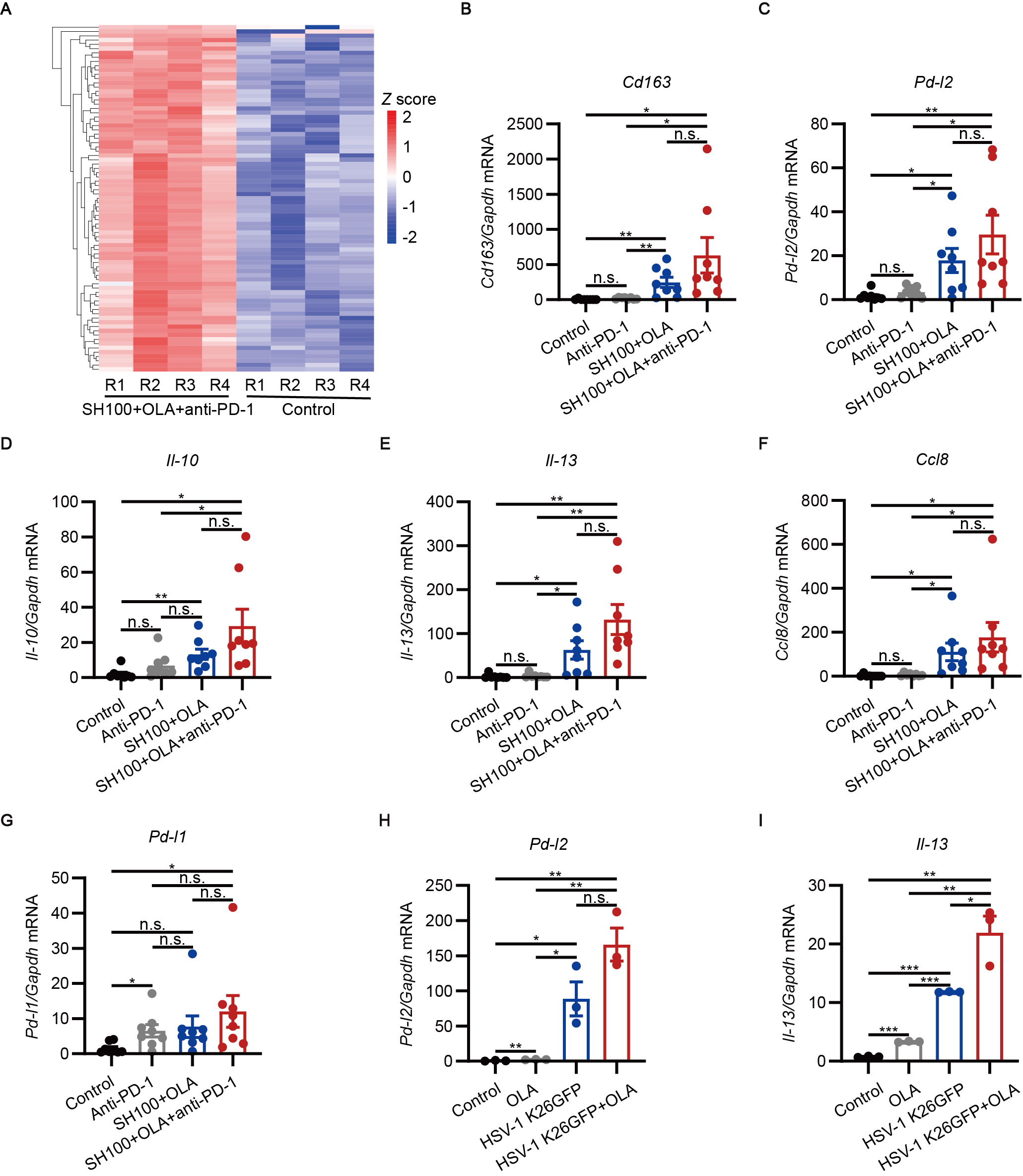


**Fig. S9. Increased immunosuppressive genes within primary tumors. A** Heat map showed 81 genes that were upregulated in the triple therapy group as compared with control group. R1, R2, R3, and R4 represent distinct biological replicates. **B-G** Primary tumors treated with the same schedule as Fig. S8A were analyzed by qPCR for indicated gene expression (*n*=8 mice per group). **H, I** qPCR analysis of 4T1 cells (*n*=3). The cells were added with 100 µM OLA for 12 h and infected with HSV-1 K26GFP (MOI=0.8) for an additional 24 h before extracting RNA. *P* values were obtained by unpaired two-tailed *t* test (**B-I**), n.s., non-significant; ^*^*P*<0.05; ^**^*P*<0.01; ^***^*P*<0.001. Data were shown as the means ± SEM.


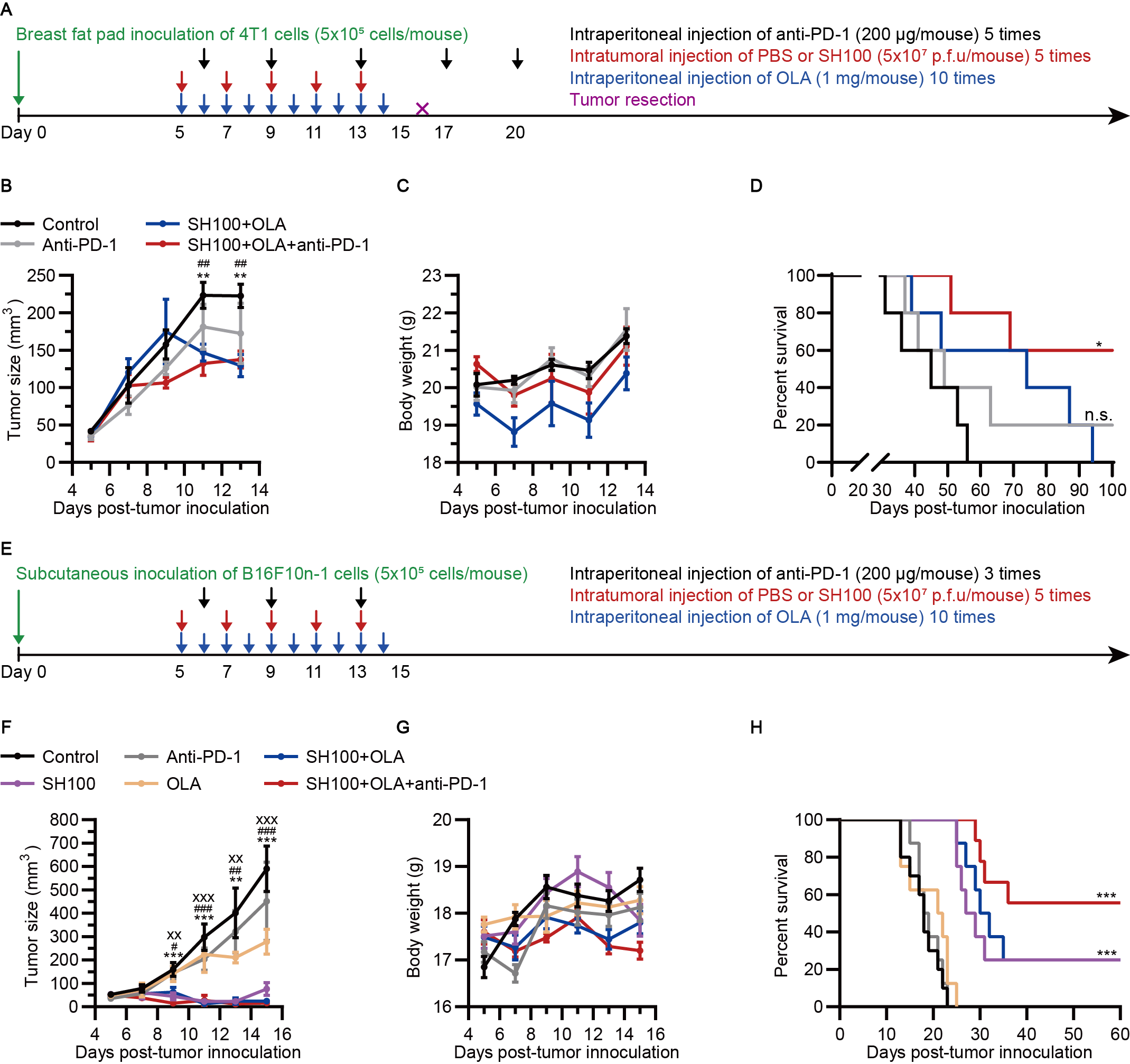


**Fig. S10. Triple therapy with OV, Olaparib, and PD-1 inhibitor enhanced antitumor efficacy. A** Illustration of optimized protocol for triple therapy in 4T1 tumors. **B-D** Evaluation the efficacy of optimized triple therapy in 4T1 tumors. Tumor growth curves (**B**); body weight (**C**); Kaplan-Meier survival curves of BALB/c mice (**D**) (*n*=5 per each group). *P* values were obtained by unpaired two-tailed *t* test (**B**), ^**, ##^*P*<0.01; or Mantel-Cox test (**D**), n.s., non-significant, ^*^*P*<0.05. Data were shown as the means ± SEM. In **B**, the symbol “^*^” denotes the difference between Control and SH100+OLA+anti-PD-1. The symbol “^#^” denotes the difference between Control and SH100+OLA. **E** Schematic illustration of the work plan in B16F10n-1 tumors. **F-H** Evaluation of the efficacy of triple therapy in B16F10n-1 tumors. Tumor growth curves (**F**); body weight (**G**); Kaplan-Meier survival curves of C57BL/6J mice (**H**). Control, *n*=10, OLA, anti-PD-1, SH100, SH100+OLA, *n*=8, SH100+OLA+anti-PD-1, *n*=9. *P* values were obtained by unpaired two-tailed *t* test (**F**), ^#^*P*<0.05; ^**, ##^*P*<0.01; ^***, ###^*P*<0.001; or Mantel-Cox test (**H**), n.s., non-significant, ^***^*P*<0.001. In **F**, the symbol “^*^” denotes the difference between Control and SH100+OLA+anti-PD-1. The symbol “^#^” denotes the difference between Control and SH100+OLA. The symbol “^x^” denotes the difference between Control and SH100. Data were shown as the means ± SEM.


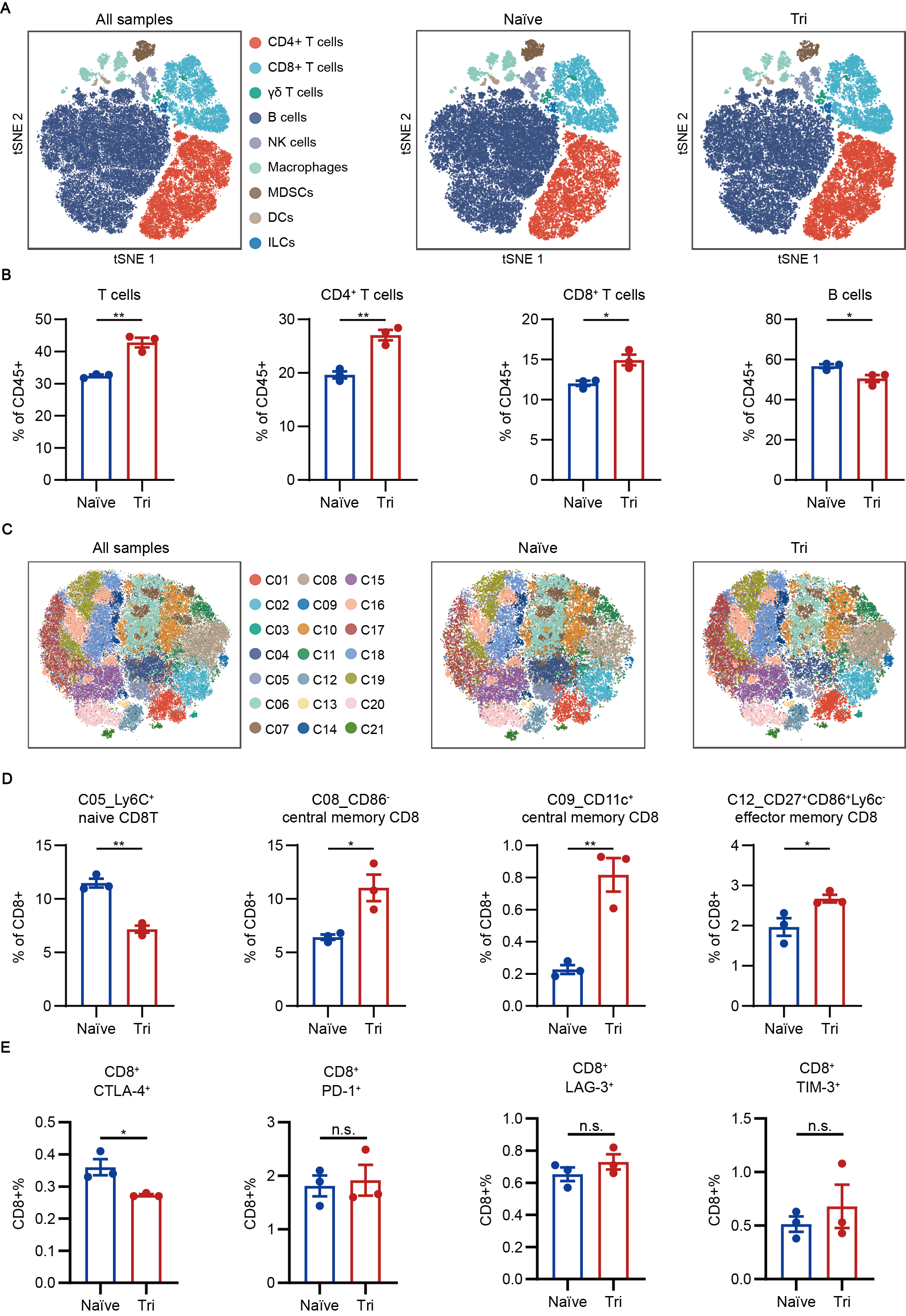


**Fig. S11. Combination therapy established long-term tumor-specific immunological memory and systemic antitumor immunity.** **A** tSNE view of all immune cells in spleen by CyTOF test from naïve group and triple group 14 days after AT3 cells re-inoculation (*n*=3 mice per group). **B** Quantification of T cells, CD4^+^ T cells, CD8^+^ T cells and B cells in (**A**). **C** tSNE view of CD8^+^ T cells in (**A**). **D** Quantification of naïve, central memory and effector memory CD8^+^ T cells. **E** Flow cytometry analysis of the expression of CTLA-4, PD-1, LAG-3 and TIM-3 of CD8^+^ T cells. *P* values were obtained by unpaired two-tailed *t* test (**B, D, E**), n.s., non-significant; ^*^*P*<0.05; ^**^*P*<0.01. Data were shown as the means ± SEM.


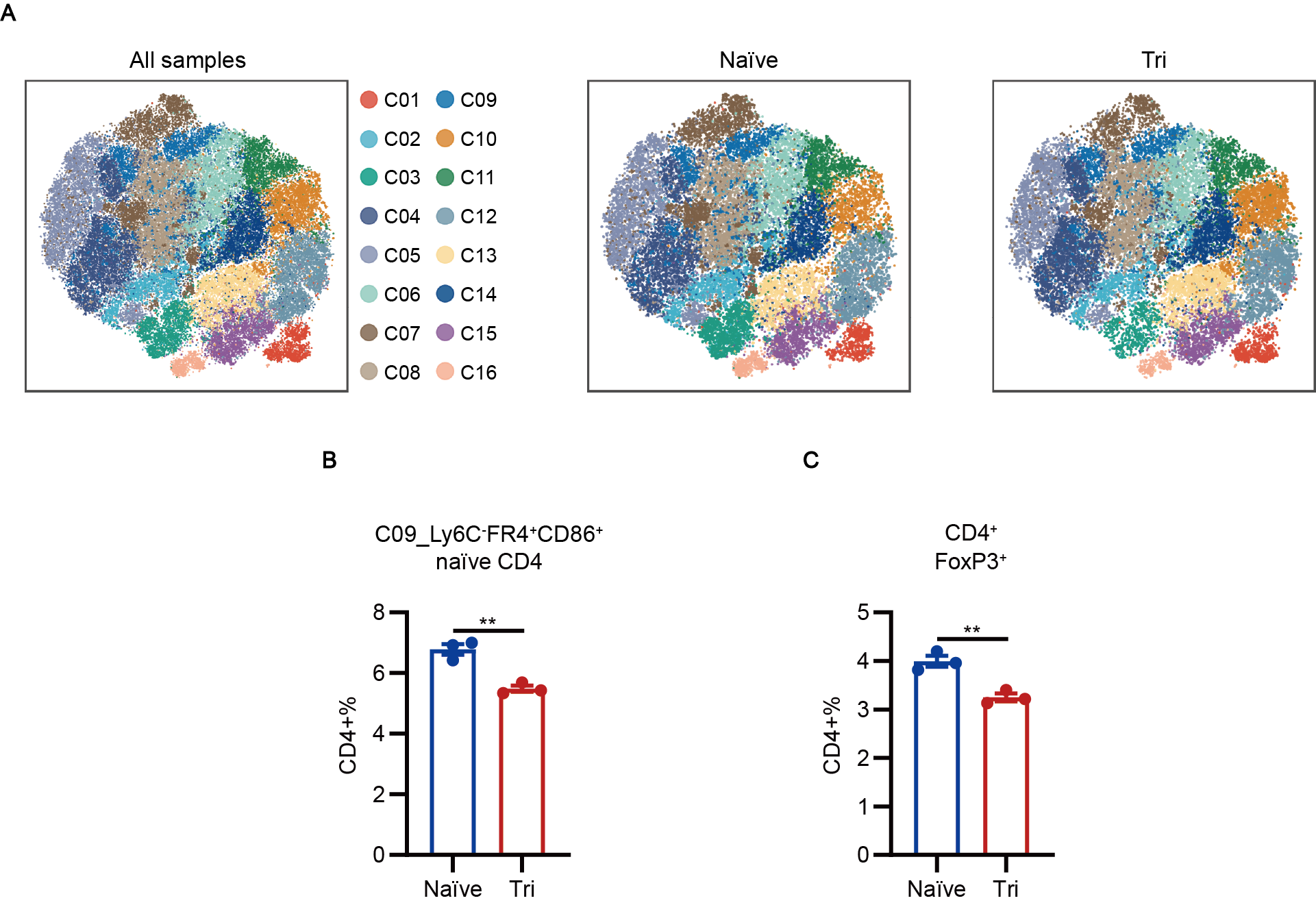


**Fig. S12. Overview of B cells in spleen by CyTOF test. A** tSNE view of CD4^+^ T cells in Fig. S11A. **B**, **C** Qualification of naïve CD4^+^ T cells (**B**) and CD4^+^ Treg (**C**). *P* values were obtained by unpaired two-tailed *t* test (**B**, **C**), ^**^*P*<0.01. Data were shown as the means ± SEM.


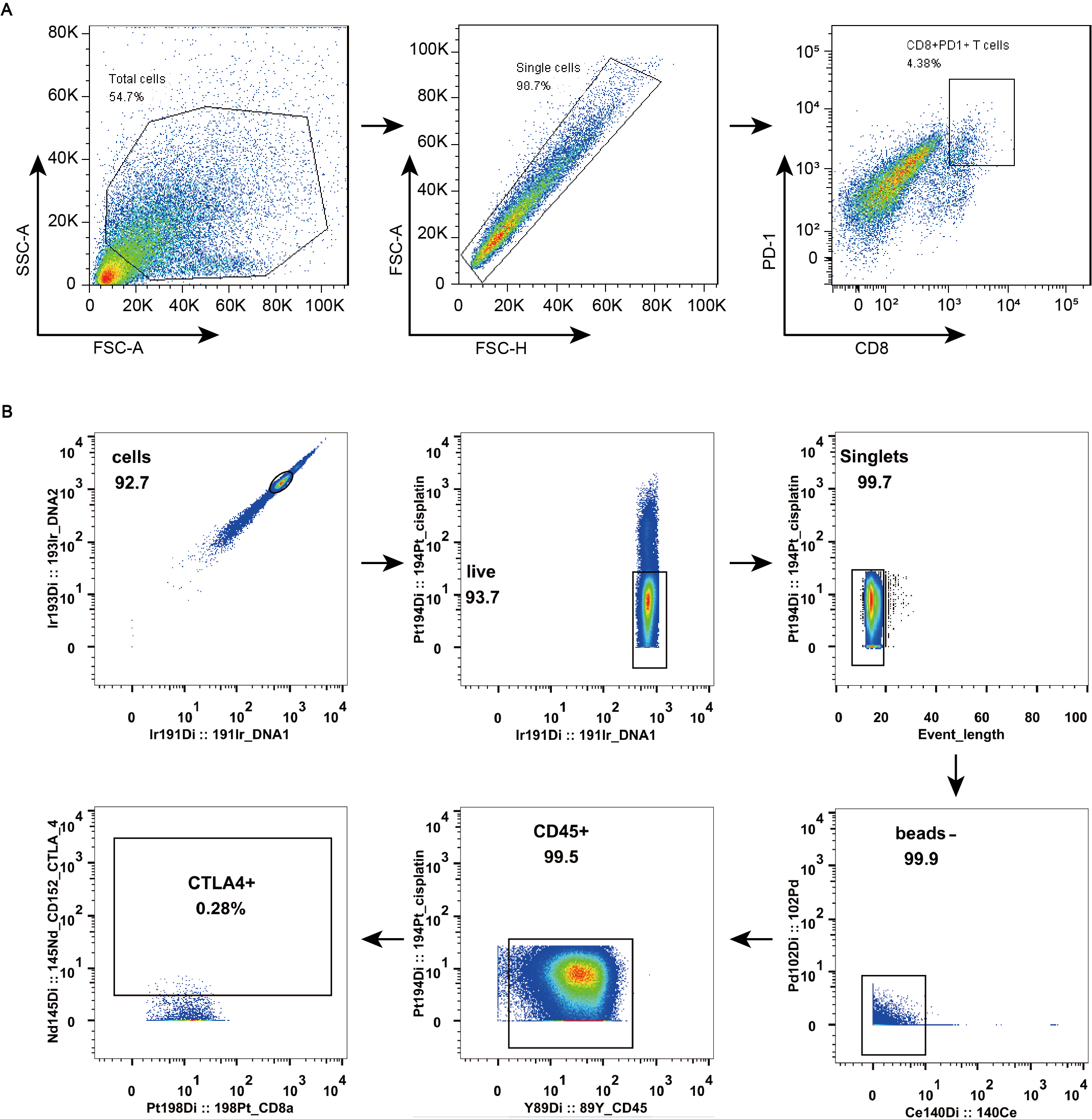


**Fig. S13. Gating strategy for detecting the specific cell types. A** Total cells from one of the samples of SH100+OLA+anti-PD-1 group in Fig. S8 were used to show the gating strategy to detect the specific cell types. **B** CyTOF gating scheme to show the immune cell populations and specific cell types in Fig. S11, S12.
